# Proteotoxic Stress Induces Phosphorylation of p62/SQSTM1 by ULK1 to Regulate Selective Autophagic Clearance of Protein Aggregates

**DOI:** 10.1371/journal.pgen.1004987

**Published:** 2015-02-27

**Authors:** Junghyun Lim, M. Lenard Lachenmayer, Shuai Wu, Wenchao Liu, Mondira Kundu, Rong Wang, Masaaki Komatsu, Young J. Oh, Yanxiang Zhao, Zhenyu Yue

**Affiliations:** 1 Department of Neurology and Neuroscience, Friedman Brain Institute, Icahn School of Medicine at Mount Sinai, New York, New York, United States of America; 2 Department of Applied Biology and Chemical Technology, State Key Laboratory of Chirosciences, The Hong Kong Polytechnic University, Hung Hom, Kowloon, Hong Kong, China; 3 Department of Pathology, St. Jude Children’s Research Hospital, Memphis, Tennessee, United States of America; 4 Department of Genetics and Genomic Sciences, Icahn Institute for Genomics and Multiscale Biology, Icahn School of Medicine at Mount Sinai, New York, New York, United States of America; 5 Department of Biochemistry, Niigata University, Niigata-shi, Japan; 6 Department of System Biology, Yonsei University College of Life Science and Biotechnology, Seoul, South Korea; University of California, Los Angeles, UNITED STATES

## Abstract

Disruption of proteostasis, or protein homeostasis, is often associated with aberrant accumulation of misfolded proteins or protein aggregates. Autophagy offers protection to cells by removing toxic protein aggregates and injured organelles in response to proteotoxic stress. However, the exact mechanism whereby autophagy recognizes and degrades misfolded or aggregated proteins has yet to be elucidated. Mounting evidence demonstrates the selectivity of autophagy, which is mediated through autophagy receptor proteins (e.g. p62/SQSTM1) linking autophagy cargos and autophagosomes. Here we report that proteotoxic stress imposed by the proteasome inhibition or expression of polyglutamine expanded huntingtin (polyQ-Htt) induces p62 phosphorylation at its ubiquitin-association (UBA) domain that regulates its binding to ubiquitinated proteins. We find that autophagy-related kinase ULK1 phosphorylates p62 at a novel phosphorylation site S409 in UBA domain. Interestingly, phosphorylation of p62 by ULK1 does not occur upon nutrient starvation, in spite of its role in canonical autophagy signaling. ULK1 also phosphorylates S405, while S409 phosphorylation critically regulates S405 phosphorylation. We find that S409 phosphorylation destabilizes the UBA dimer interface, and increases binding affinity of p62 to ubiquitin. Furthermore, lack of S409 phosphorylation causes accumulation of p62, aberrant localization of autophagy proteins and inhibition of the clearance of ubiquitinated proteins or polyQ-Htt. Therefore, our data provide mechanistic insights into the regulation of selective autophagy by ULK1 and p62 upon proteotoxic stress. Our study suggests a potential novel drug target in developing autophagy-based therapeutics for the treatment of proteinopathies including Huntington’s disease.

## Author Summary

Accumulation of misfolded proteins deposited in the form of inclusion bodies is a common pathological hallmark for many human genetic diseases, particularly for the neurodegenerative disorders. The aggregation of the disease related proteins suggests a failure of the cellular machineries that maintain the protein homeostasis or proteostasis. The cellular clearance pathways, e.g. autophagy-lysosomal pathway, may not be of high efficiency in the face of rapid formation of misfolded protein aggregates. Thus, understanding of intrinsic mechanism whereby autophagy offers protection to cells by removing toxic protein aggregates is important. Here we report that a signaling transduction event that chemically modifies autophagy receptor protein p62/SQSTM1 regulates the receptor’s binding affinity to small molecule called ubiquitin(essential for marking the protein for degradation), as well as the selective degradation of targeted proteins. Furthermore, we find that expression of Huntington’s disease (HD) associated protein aggregates (containing polyglutamine or polyQ expansion) triggers the same modification of p62, which is dependent on the length of the polyQ expansion, suggesting a protective response of the cell by activating autophagy toward degradation of toxic aggregates. The modification of p62 also occurs in HD model brains in an age-dependent manner. Our study sheds light on the regulation of selective autophagy and provides a rationale for targeting p62 modification to treat aggregate diseases including HD.

## Introduction

Protein homeostasis, or proteostasis, is controlled by cellular pathways responsible for protein synthesis, folding, trafficking and degradation. Understanding the cellular functions that maintain proteostasis is central to the elucidation of the disease mechanisms associated with protein misfolding and aggregation. Autophagy is a cell catabolic pathway that, through the formation of autophagosomes, sequesters and delivers cytosolic cargos to lysosomes for degradation. Autophagy occurs constitutively in almost every cell type and plays an important role in the prevention of ubiquitinated protein overflow/aggregation [1]. Autophagy is up-regulated in response to cellular stresses such as nutrient starvation, hypoxia, growth factor withdrawal, endoplasmic reticulum(ER) stress, and pathogen infection [2]. Autophagy activity can be increased to compensate for the deficiency of the ubiquitin proteasome system(UPS) and alleviate subsequent proteotoxic stress [3]. However, the regulatory mechanisms remain largely elusive.

The ULK1/Atg1 complex, consisting of ULK1 kinase, ATG13, FIP200 and ATG101, is required for the initiation of autophagy [4]. ULK1 is a mammalian homolog of the *C. elegans* uncoordinated 51 serine/threonine protein kinase [5] and its activity is regulated by mTOR and AMPK in response to nutrient availability [6]. ULK1 controls autophagy activity by phosphorylating multiple substrates, such as FIP200, ATG13, Beclin 1, AMPK, Ambra1, ATG9 and FUNDC1 [7–14]. ULK1 kinase activity is upregulated in response to hypoxia and is required for hypoxia-induced autophagy activation [15].

Emerging evidence indicates that autophagy has selectivity in substrate degradation, a process called selective autophagy, which is mediated by a specific group of autophagy receptors [16]. p62/SQSTM1(Sequestosome-1) is a prototypical autophagy receptor that recognizes ubiquitinated cargos and tethers them to the autophagy machinery by direct binding to microtubule-associated protein light chain 3(LC3, a mammalian Atg8 homolog) [17], enabling the degradation of selective substrates [18]. Additional autophagy receptors have been identified(e.g. NBR1 and ALFY) later and they are expected to have similar functions or collaborate with p62 during selective autophagy [19–21]. It is believed that p62 plays a fundamental role in the clearance of protein aggregates [17,22], damaged mitochondria [23], midbody rings [24], peroxisomes [25] and invading microbes [26] through selective autophagy. However, the molecular and structural characteristics underlying the precise function of p62 in this pathway are poorly understood. Previous reports suggested that phosphorylation of p62 at S405 by casein kinase 2(CK2) or TBK-1 may regulate the clearance of expanded polyglutamine(polyQ) proteins or mycobacteria, respectively [27,28]; Interestingly, a recent report implicates ULK1 in this phosphorylation of p62 under oligomycin-induced metabolic stress [29], but whether phosphorylation of p62 occurs in response to disease proteins and the molecular basis of p62 phosphorylation in regulating cargo recognition has yet to be shown.

Here we report that proteotoxic stress triggers phosphorylation of p62 at multiple sites in its ubiquitin association(UBA) domain. We find that accumulation of protein aggregates, such as polyubiquitinated proteins(due to proteasome inhibition) or polyQ-expanded proteins, induce the interaction of p62 with ULK1 and ULK1-dependent p62 phosphorylation in its UBA domain. Phosphorylation of a novel site S409 regulates the dimer interface of p62’s UBA domain and enhances the affinity of p62 to ubiquitin, while lack of the phosphorylation impairs the recruitment of autophagy proteins and degradation of ubiquitinated proteins or polyQ-expansion proteins. Our study thus reveals that selective autophagy can be triggered by ULK1-dependent p62 phosphorylation, and that this event regulates ubiquitinated protein or aggregate-prone disease protein clearance.

## Results

### Identification of S409 in p62 UBA domain as a novel phosphorylation site by ULK1

**Fig 1:**
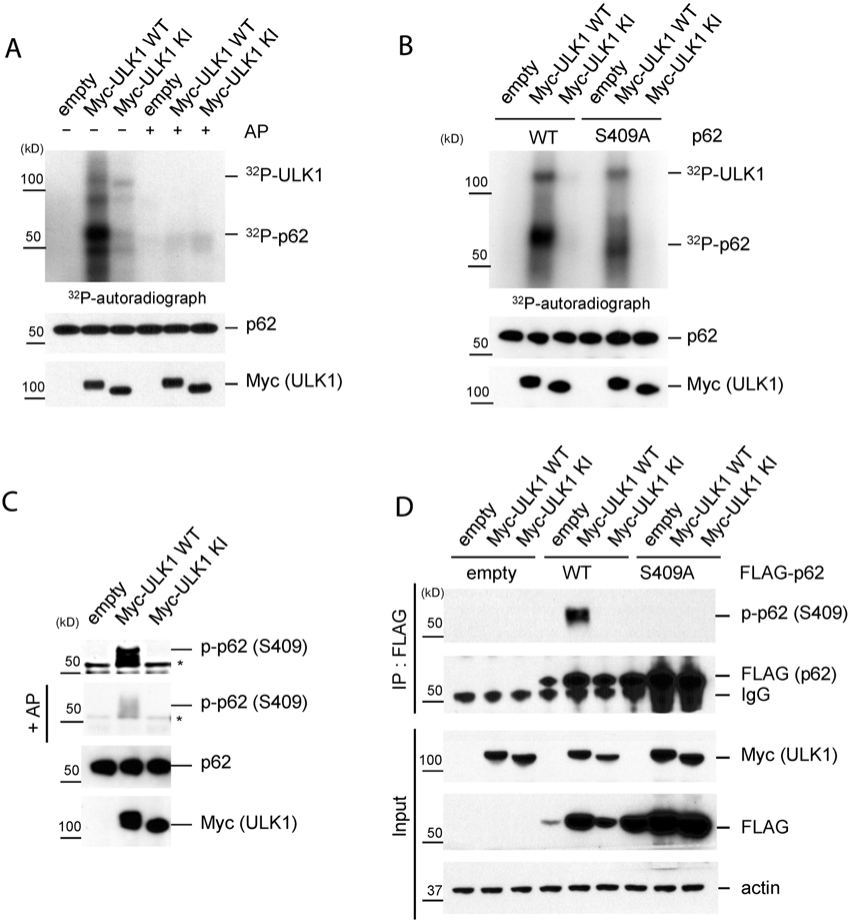
ULK1 phosphorylates p62 at S409. **A.-C**. *In vitro* p62 phosphorylation assay by ULK1 with purified MBP-p62 WT or mutant proteins. Bacterially expressed MBP-p62 was purified and then MBP tag was cleaved by Factor Xa. The purified p62 was incubated with Myc-ULK1 WT or KI mutant IPed from transfected HEK293T cells at 37°C for 30 min. Phosphorylation of p62 was examined by ^32^P-labeling and autoradiography or p-S409 specific antibody. **A**. p62 is an ULK1 substrate *in vitro*. Alkaline phosphatase(AP) was used to dephosphorylate p62. ^32^P-autoradiograph shows autophosphorylation of ULK1 and p62 phosphorylation. **B**. ULK1 phosphorylates p62 at Ser409 *in vitro*. Purified MBP-p62 WT or S409A proteins were used in ULK1 kinase assay in the presence of ^32^P-ATP. **C**. ULK1 phosphorylates Ser409 of p62. Purified MBP-62 WT proteins were incubated with Myc-ULK1 variants isolated from transfected HEK 293T cells. Immunoblotting assay with indicated antibodies, including phospho-p62 antibody against Ser409, was followed. Afterwards, the membrane probed with p-p62 antibody(S409) was incubated with alkaline phosphatase(AP) to dephosphorylate p62. Asterisks indicate nonspecific bands. **D**. p62 S409 is a ULK1 substrate. HEK 293T cells were transfected wit empty vector, FLAG-p62 WT or FLAG-p62 S409A together with Myc-ULK1 WT or KI. IP with anti-FLAG antibody was performed, followed by Westernblot assay with indicated antibodies.

A previous report suggested a link of p62 and ULK1, prompting us to test p62 as a potential ULK1 substrate [30]. We thus performed an *in vitro* phosphorylation assay with purified MBP-tagged p62 proteins and immune-isolated Myc-tagged ULK1 wild type(WT) or kinase inactive(KI) mutant [31] in the presence of ^32^P-ATP. ULK1 WT, but not KI, was able to phosphorylate p62 *in vitro*, while the labeling was abolished with dephosphorylating alkaline phosphatase(AP)(Fig. 1A). To map the putative phosphorylation site/s, we repeated the same assay using various purified truncation mutants of p62 (S1A, B, C Fig.) [32]. While M1 mutant(PB1 domain deletion) retained the evident ^32^P-labeling in the presence of ULK1 WT, M4(UBA domain deletion) or M7(both PB1 and UBA domain deletion) showed reduced ^32^P-labeling. The result suggests that the UBA domain contains primary ULK1 phosphorylation sites. To precisely map the residues phosphorylated by ULK1, we performed mass spectrometry analysis with immuno-isolated FLAG-p62 from HEK293T cells transfected with ULK1 WT. Our analysis identified serine 409 in p62 UBA domain as a potential phosphorylation site (S1D Fig.) To validate S409 as the target site, we substituted serine 409 with alanine to generate a phosphorylation-null mutant(S409A), purified MBP-p62-S409A proteins and performed an *in vitro* phosphorylation assay. In agreement with the mass spectrometry results, ULK1-dependent phosphorylation of the S409A mutant was markedly reduced compared to p62 WT (Fig. 1B), indicating that S409 is a ULK1 substrate. Residual ^32^P-labeling in S409A may suggest that ULK1 phosphorylates additional sites in p62.

To investigate S409 phosphorylation event in cells, we raised a phosphorylation-specific antibody against phosphorylated S409 of p62(p-S409) and validated its specificity. Incubation of purified p62 with ULK1 WT(but not KI mutant) results in the detection of a strong signal with anti-p-S409 antibody (Fig. 1C), and incubation with phosphatase abolished this band. To further validate the specificity of anti-p-S409 antibody, we immunoprecipitated(IPed) FLAG-p62 or-S409A in the presence of overexpressed Myc-ULK1 WT or KI from transfected HEK 293T cells, followed by anti-p-S409 antibody detection of the phosphorylation. We observed a strong signal only in the sample co-expressing FLAG-p62 WT and Myc-ULK1 WT (Fig. 1D). Collectively, our data indicate that S409 of p62 is a novel ULK1 kinase substrate *in vitro*.

### Proteasome inhibition, but not nutrient starvation, induces p62 phosphorylation at S409

**Fig 2:**
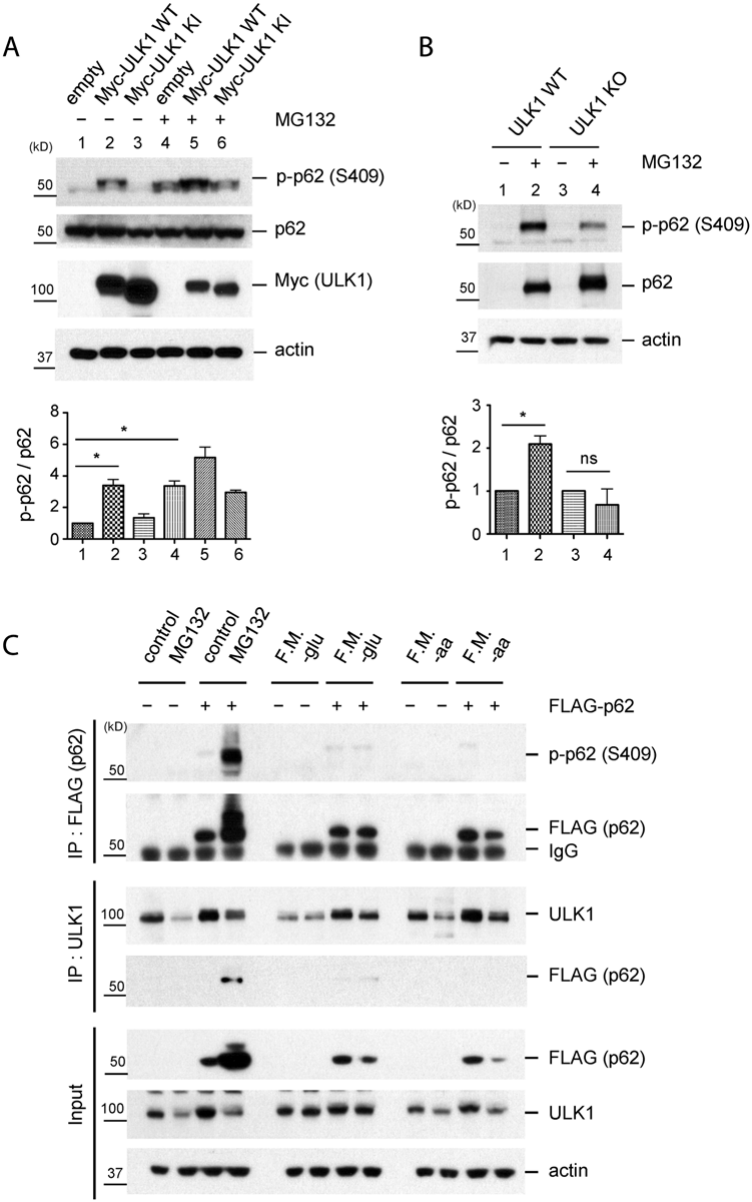
Phosphorylation of p62 at S409 is enhanced upon proteasome inhibition. **A**. ULK1 mediates p-S409 of p62 upon MG132 treatment. HEK 293T cells transfected with empty vector, Myc-ULK1 WT or KI were treated with MG132 and then cell lysates were analysed with indicated antibodies(Top). The ratio of p-p62 and p62 was obtained by dividing the level of p-p62 by total p62(n = 3). One sample *t*-test was used and data are represented as mean ± SEM(n = 3). * *p* < 0.05; ns, not significant(bottom). **B**. ULK1 is important for MG132-induced p-S409 of p62. WT and ULK1 KO MEFs were treated with MG132 and analyzed with indicated antibodies(Top). The ratio of p-p62 and p62 was obtained by dividing the level of p-p62 by total p62(n = 3). One sample *t*-test was used and data are represented as mean ± SEM(n = 3). * *p* < 0.05; ns, not significant(bottom). **C**. MG132 treatment but not starvation increases p-S409 levels of p62 or ULK1-p62 interaction. p62 KO MEFs stably expressing empty vector or FLAG-p62 were incubated with MG132 or starved for glucose(-glu) or amino acid(-aa). F.M. indicates full medium as a control. Subsequently, IP with anti-FLAG or-ULK1 antibodies were performed and immunoprecipitants were analyzed with indicated antibodies.

Based on the previous report on the occurrence of p62 phosphorylation at S405 upon MG132 treatment, a proteasome inhibitor causing accumulation of polyubiquitinated proteins and p62 [27], we first tested whether accumulation of polyubiquitinated proteins would lead to ULK1-mediated phosphorylation of p62 at serine 409. p62 S409 phosphorylation was induced by the exposure of HEK293T cells to MG132 (Fig. 2A). This effect was also observed by overexpressing WT ULK1, but not a KI form of this kinase in the absence of MG132. Although MG132 treatment led to higher levels of total p62 protein, p-S409 upon MG132 treatment was severely impaired in ULK1 KO MEFs (Fig. 2B). The incomplete loss of p-S409 in the absence of ULK1 suggests that other kinases(e.g. ULK2, a homolog of ULK1) could alternatively phosphorylate S409. To test the redundant role of ULK1 and ULK2 in p62 phosphorylation, we studied ULK1/2 double knockout(DKO) MEFs treated with MG132 (S2A Fig.). Deletion of both ULK1 and ULK2 completely abolished p-S409 signal upon MG132 treatment, suggesting that ULK1 and ULK2 are the major kinases that phosphorylate p62 at S409 under this condition. These results altogether indicate that p62 is phosphorylated at S409 in response to proteotoxic stress, at least in part, by ULK1, although we cannot rule out that CK2 or TBK1 might also phosphorylate S409, similar to S405 [27,28].

Using p62 KO MEFs stably transfected with FLAG-p62 WT or control empty vector, we performed IP with anti-FLAG or-ULK1 antibodies after MG132 treatment. We detected a clear p-S409 signal along with enhanced ULK1-p62 interaction in MG132-treated cells, compared to normal medium condition (Fig. 2C). Since ULK1 regulates autophagy initiation by phosphorylating several autophagy related proteins in response to nutrient starvation [6,9] and p62 also plays a role in the early stage autophagosome formation [30], we investigated if nutrient starvation induces ULK1-p62 interaction and/or increases p-S409 levels. In contrast to proteasome inhibition, neither glucose withdrawal nor amino acid starvation induces p62 phosphorylation at S409 or interaction between p62 and ULK1 (Fig. 2C), suggesting a specific effect of the proteasome inhibition(associated with accumulation of protein aggregates) in S409 phosphorylation.

### PolyQ-Htt expression induces p62 phosphorylation at S409 in a polyQ length-dependent manner

**Fig 3:**
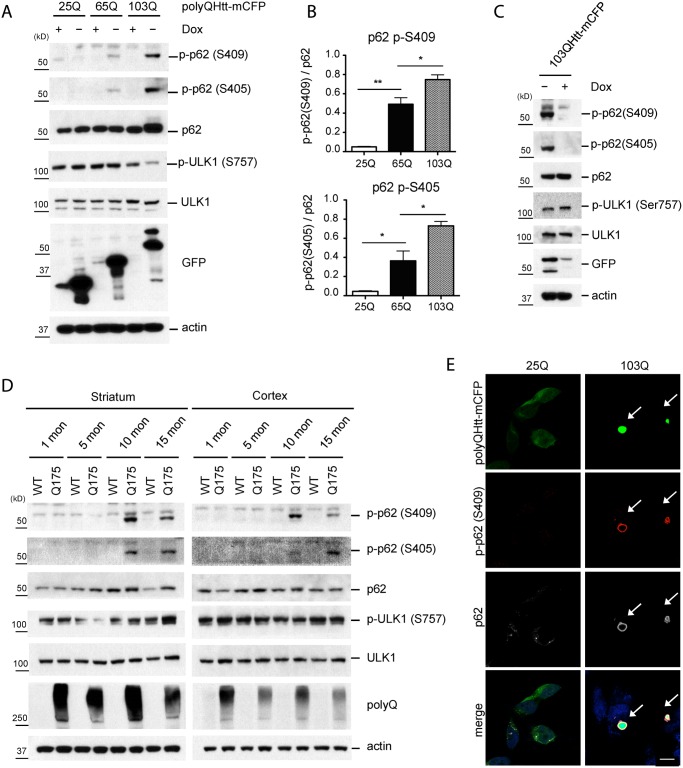
Phosphorylation of p62 at S409 is enhanced upon expression of polyQ-Htt proteins. **A**. Expanded polyQ-mCFP induces p-S405 and p-S409 of p62 in a polyQ length dependent manner. HeLa/25Q, 65Q, 103Q—mCFP cells were cultured with or without doxycycline to regulate the expression of each protein. Cellular lysates were assayed with indicated antibodies. GFP antibody was used to monitor induction of polyQ-mCFP proteins. **B**. The ratio for Fig. 3A was obtained by dividing the level of p-p62 by total p62(n = 3). One sample *t*-test and student *t*-test were used and data are represented as mean ± SEM(n = 3). * *p* < 0.05; ** *p* < 0.01; ns, not significant **C**. Shutoff of the expression of 103Q-mCFP reduces p-S405 and p-S409 of p62. Induced HeLa/103Q-mCFP cells were replenished with Doxycycline to turn off the protein expression and then were analyzed with indicated antibodies. **D**. p62 phosphorylation is increased in z_Q175 HD mouse model. Striatal and cortical lysates of 1, 5, 10, and 15 months old WT and Q175 mice were prepared and analyzed with indicated antibodies(n = 4). **E**. P-S409 localizes to and surrounds 103Q-mCFP positive inclusions. Induced HeLa/25Q-mCFP and HeLa/103Q-mCFP cells were stained with anti-p62 and p-p62(S409) and examined under confocal microscope. Scale bar = 10 μm.

To further understand the physiologic significance of ULK1-mediated p62 phosphorylation, we next examined whether expression of aggregate-prone disease proteins would induce p62 S409 phosphorylation. We employed an inducible HeLa/polyQ-mCFP cell line, which expresses an mCFP-tagged polypeptide encoding the first 17 amino acids of huntingtin with a polyQ expansion under control of a doxycycline-responsive promoter(Tet-off system) [33]. The expanded trinucleotide(CAG) tract in exon 1 of the *huntingtin* gene is the major cause of Huntington’s disease(HD) [34] and particularly, CAG repeats beyond 35 in number are known to increase disease risk. Since protein aggregation correlates with the length of polyQ tract in these cases, we tested three different lengths of polyQ tract: 25Q, 65Q and 103Q. Notably, while induction of a non-toxic form of 25Q failed to induce p-S409, expression of the toxic species 65Q- and 103Q-mCFP triggered robust p62 phosphorylation at S409, whose abundance correlated with the length of polyQ (Figs. 3A, 3B). The phosphorylation coincided with ULK1 activation upon 103Q expression, evidenced by the reduction of a known inhibitory modification(S757 phosphorylation) in this kinase (Fig. 3A) [6]. The induction of p62 S409 phosphorylation by 65Q- and 103Q-mCFP was accompanied by S405 phosphorylation(equivalent to human S403), a modification previously reported [27,28,35]. Importantly, both S409 and S405 phosphorylation were dependent on the expression of polyQ tracts, since addition of doxycycline that represses the expression of polyQ expansion proteins resulted in loss of the phosphorylated form (Fig. 3C). These results indicate that both S405 and S409 phosphorylation are specific responses to the accumulation of polyQ aggregates.

To confirm the physiologic relevance of these observations, we examined the brain lysates of z_Q175 HD mice, which carry a knock-in allele of approximately 175 CAG repeats [36]. Immunoblot analysis detected p62 phosphorylation at both S405 and S409 in the striatum and cortex of 10 and 15 months old animals, but not younger(1 or 5 months old) animals. These observations confirm that the phosphorylation of p62 UBA domain at S405 and S409 also occurs *in vivo* in response to the accumulation of polyQ-expansion protein(Htt), in an age-dependent manner (Fig. 3D). IF staining with the anti-p-S409 antibody primarily labeled aggregate structures by surrounding the 103Q-CFP inclusions. In contrast, the absence of aggregates in 25Q-CFP cells coincided with the lack of the p-S409 signal and redistribution of p62 in small puncta (Fig. 3E). This observation was in agreement with the immunoblot analysis and suggested the involvement of p-S409 in the recognition of expanded polyQ inclusions.

*Atg7* conditional KO mouse is another validated disease model of accumulation of ubiquitinated proteins [1,37]. We examined brain lysates of two different *Atg7* conditional KO mice(*Atg7* ^f/f^;Syn-Cre and *Atg7* ^f/f^;Nes-Cre) and found marked accumulation of total p62 as well as p-S409 but not in control brains. It is likely due to an arrest of degradation of p62 with p-S409 when autophagy is inactivated (S2B Fig.). p62 and p62-p-S409 was absent in *Atg7* and *p62* double-KO mouse brains (S2C Fig.), confirming the specificity of anti p-S409 antibody and the occurrence of p62 p-S409 in tissue, perhaps due to ULK1—p62 signaling as a response to the ubiquitinated protein accumulation. A similar result was observed with anti-p-S405 antibody in *Atg7* ^f/f^;Syn-Cre or *Atg7* ^f/f^;Nes-Cre versus control brains (S2B, C Figs.). Taken together, the results support the idea that ULK1-mediated S409 phosphorylation in p62 is a specific response to the expression of aggregate-prone disease proteins.

### Accumulation of protein aggregates induces the interaction of ULK1 and p62/SQSTM1

**Fig 4:**
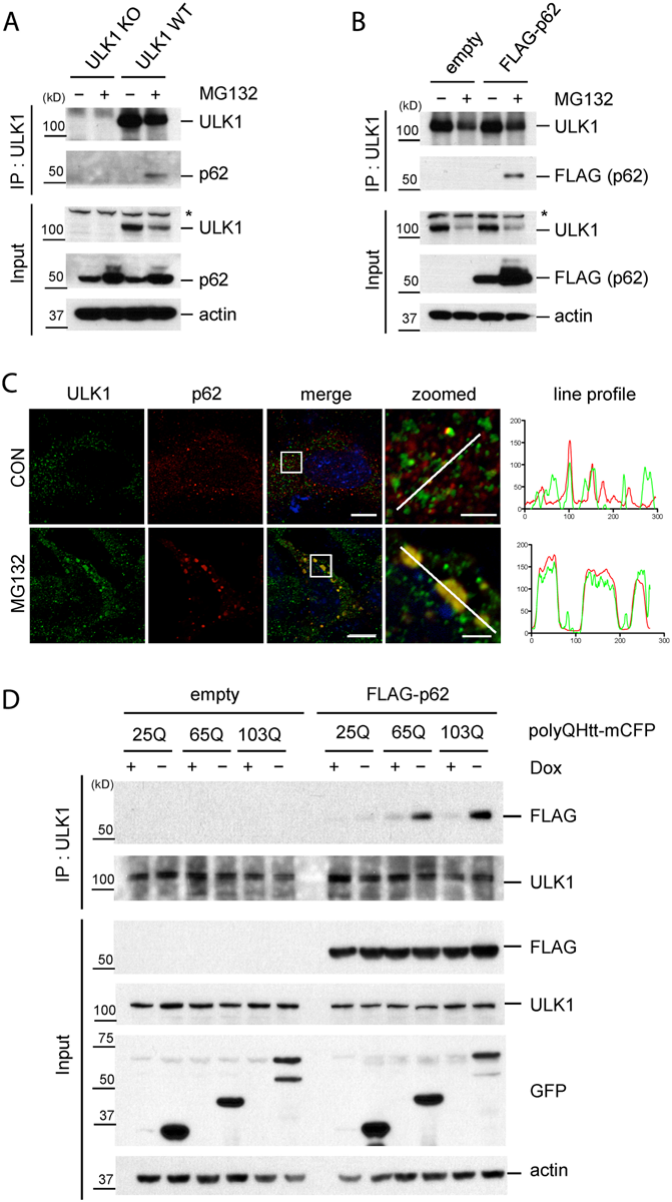
Accumulation of protein aggregates induces the interaction between ULK1 and p62. **A**. The accumulation of ubiquitinated proteins induces ULK1-p62 interaction. WT and ULK1 KO MEFs lysates treated with or without MG132 were subjected to IP with anti-ULK1 antibody and immunoblotted with anti-ULK1 and-p62 antibodies. Asterisks indicate non-specific bands. **B**. ULK1-p62 interaction in a p62 stable cell line. p62 KO MEFs stably expressing FLAG-p62 or empty vector were incubated with or without MG132. IP was performed with anti-ULK1 antibody, followed by immunoblot assay using indicated antibodies. **C**. Co-localization of p62 and ULK1 upon MG132. p62 KO MEFs stably expressing FLAG-p62 WT were treated with MG132, fixed, stained with ULK1(green) and p62(red) antibodies, and visualized under fluorescent microscope. Line profile was used to illustrate co-localization between p62 and ULK1. Green and red lines indicate ULK1 and p62 staining profiles, respectively. Scale bar = 10 μm and 2.5 μm for zoomed images. **D**. ULK1-p62 interaction increases upon induction of expanded polyQ proteins. HeLa/25Q, 65Q, 103Q—mCFP cells were transfected with empty vector or FLAG-p62 and were cultured with or without doxycycline to regulate the expression of each protein. Cellular lysates were used to perform IP with anti-ULK1 antibody and immunoblot assay with indicated antibodies was followed. GFP antibody was used to monitor induction of polyQ-mCFP proteins.

Since we observed that p62 phosphorylation, along with the interaction of p62 and ULK1, is enhanced upon the accumulation of ubiquitinated proteins(due to MG132 treatment), but not upon nutrient starvation (Fig. 2C), we hypothesized that the enhanced interaction leads to ULK1-mediated phosphorylation of p62 at serine 409. To validate the interaction at endogenous level, we performed IP using anti-ULK1 antibody from mouse embryonic fibroblasts(MEF) cells. Under normal condition, p62 was not detected in the immunoprecipitants with an anti-p62 antibody. However, treatment with MG132 induced the interaction of the endogenous ULK1 and p62, despite the reduction of endogenous ULK1 levels (Fig. 4A). In the absence of ULK1, anti-ULK1 antibody did not pull down p62 in ULK1 knock-out(KO) MEFs [38], supporting the specificity of the interaction. We next validated the interaction by using a p62 KO MEF cells stably expressing FLAG-tagged p62(FLAG-p62). Consistently, IP experiment with anti-ULK1 antibody demonstrates the interaction of FLAG-p62 and ULK1 only under MG132 treatment, despite the reduction of endogenous ULK1 levels (Fig. 4B). Furthermore, our immunofluorescent(IF) staining showed that stably-expressed FLAG-p62 and endogenous ULK1 co-localize in a large number of puncta or protein aggregates upon MG132 treatment (Fig. 4C), in agreement with enhanced interaction between ULK1 and p62 in that condition. In contrast, ULK1 and p62 partially co-localize under normal condition in small dots, consistent with the previous observation [30].

We next investigated if the accumulation of aggregate-prone disease proteins induces interaction between p62 and ULK1. Interestingly, induction of the toxic species 65Q- and 103Q-mCFP triggered ULK1—p62 interaction; in contrast, non-toxic form of 25Q-mCFP showed no effect in enhancing ULK1—p62 interaction (Fig. 4D). Taken our data together, our study suggests that accumulation of protein aggregates induce the interaction between ULK1 and p62 and consequent phosphorylation.

### Identification of protein motifs and activities required for ULK1 and p62 interaction

To further examine the ULK1 and p62 interaction, we next mapped the sequence of p62 and ULK1 important for their interaction. First, we asked if ubiquitin binding activity of p62 is required for their interaction. We transfected FLAG-p62 WT or mutant p62 F408V mutant(impaired in ubiquitin binding) [39] and performed IP. In contrast to p62 WT, p62 F408V mutant showed a reduced interaction with ULK1 after MG132 treatment (S3A, B Figs.), indicating that the C-terminal UBA sequence motif important for ubiquitin binding is critical for ULK1 interaction. Previously, it was shown that ULK1 KI has an impaired autophosphorylation and conformational changes leading to the exposure of C-terminal domain, which shows reduced interaction with Atg13, a well-known ULK1 substrate [31]. To see if a similar mechanism is involved in ULK1 and p62 interaction, we performed IP between Myc-ULK1 WT or KI with FLAG-p62. Our data showed that ULK1 KI mutant binds poorly to p62 compared to ULK1 WT, suggesting that ULK1 interacts with p62, and their interaction depends on an intact kinase activity of ULK1 (S3C Fig.). In searching for a binding domain in ULK1, we transfected mCherry-p62 wild type(WT) plasmid together with various deletion mutants of FLAG-tagged ULK1 plasmids and then examined the interaction by IP with anti-FLAG antibody after MG132 treatment. Overexpressed ULK1 WT and p62 interact with each other; however, deletion of N-terminal kinase domain of ULK1(Δkinase) caused a reduction in binding to mCherry-p62 protein, compared to WT or other mutants (S3D Fig.), suggesting an important role for the kinase domain of ULK1 in binding p62.

### Phosphorylation of S409 enhances ubiquitin binding affinity of p62

**Fig 5:**
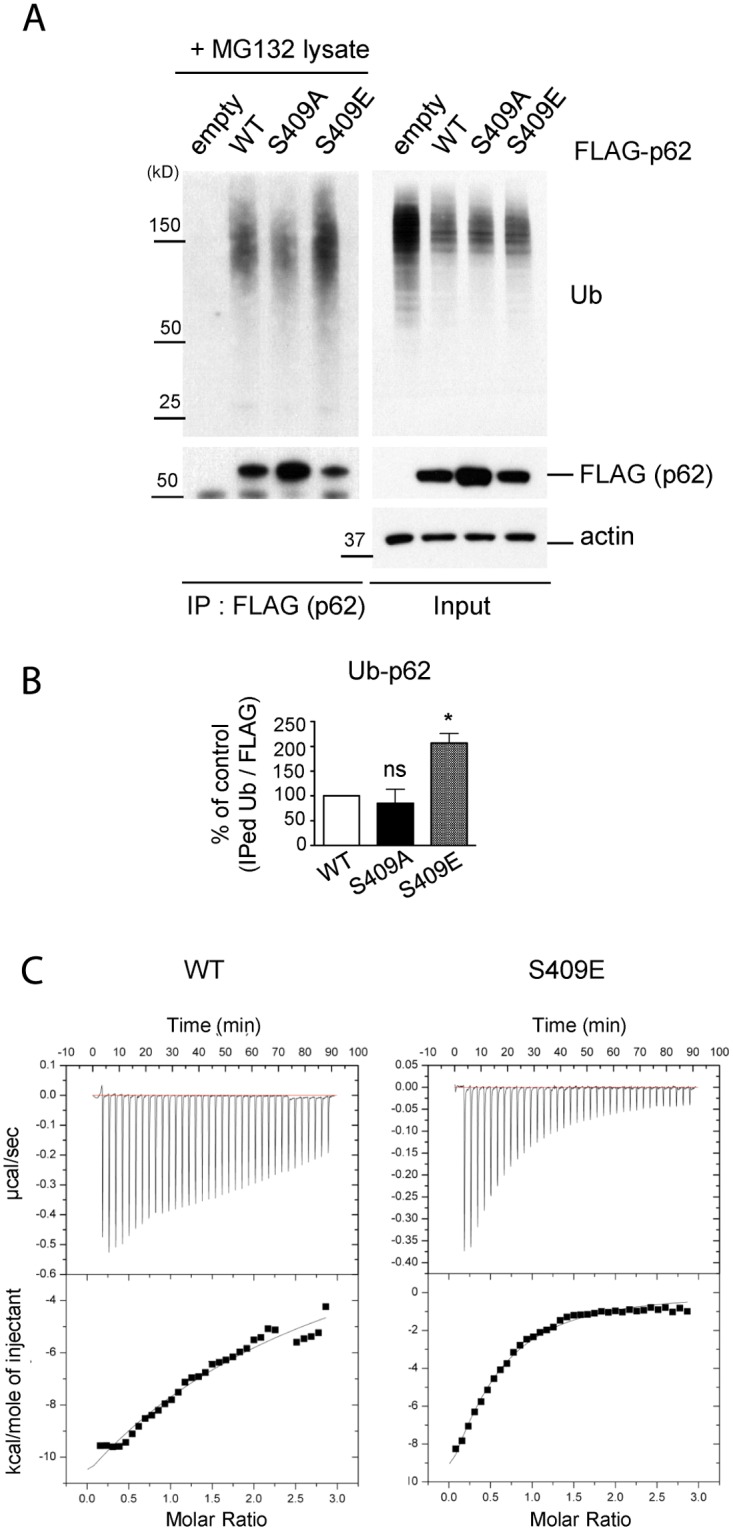
ULK1-mediated phosphorylation of p62 at S409 enhances p62 and Ub binding affinity. **A**. P-S409 enhances binding between p62 and poly-Ub proteins. Cellular lysates of p62 KO MEFs stably expressing empty vector, FLAG-p62 WT, S409A, or S409E were incubated with p62 KO MEFs lysates treated with MG132 and subsequently subjected to IP using anti-FLAG antibody. Immunoblot assay with indicated antibodies was followed. **B**. Quantification of the results from Fig. 5A were obtained by normalizing levels of IPed Ub to FLAG blots; then S409A or S409E were normalized to WT. One sample *t*-test was used and data are represented as mean ± SEM(n = 3). * *p* < 0.05; ns, not significant **C**. p62 UBA S409E has an enhanced binding affinity to mono-Ub. Binding affinities of p62 UBA WT(left) or S409E(right) to mono-Ub were measured by Isothermal Titration Calorimetry(ITC). Representative ITC profiles are shown.

S409 resides in the p62 UBA domain near S405, whose phosphorylation has been reported to enhance the p62–Ubiquitin(Ub) interaction [27]. We therefore asked whether p-S409 has any impact on the affinity of p62 for ubiquitin. To this end we used p62 KO MEF cell lines stably expressing FLAG-p62 WT, S409A or a phosphorylation-mimicking mutant S409E. To minimize the interference of p62 self-ubiquitination in this experiment, we incubated cell lysates from p62 KO MEFs treated with MG132(providing enriched polyubiquitinated proteins) and MEF lysates stably expressing FLAG-p62 variants(providing bait), followed by IP with anti-FLAG antibody. S409E pulled down significantly higher levels of polyubiquitinated(poly-Ub) proteins compared to p62 WT; in contrast, S409A pulled down the similar levels of poly-Ub proteins as WT (Figs. 5A, B). Furthermore, we examined the binding of p62 WT, S409A or S409E to K48- versus K63- linked poly-Ub chains *in vitro*. Purified MBP-p62 variants were incubated with either K48 or K63 poly-Ub chains, followed by MBP pull down. The results showed no difference between MBP-p62 variants in binding K48-linked poly-Ub in our assay, whereas S409E binds higher amounts of K63-linked poly-Ub compared to WT and S409A (S4A, S4B Figs.).

We next investigated the binding affinity of the p62 UBA to mono-Ub by performing Isothermal Titration Calorimetry(ITC) assays. The analysis indicated that the K_d_ for purified p62 UBA WT was 51.4 μM, confirming a weak interaction between p62 UBA domain and mono-Ub as previously reported (Fig. 5C, left) [40]. The K_d_ for S409E mutant, however, was 27.5 μM, suggesting an increased Ub binding by S409E (Fig. 5C, right).

### Phosphorylation of S409 destabilizes the UBA dimer interface and regulates phosphorylation of S405

**Fig 6:**
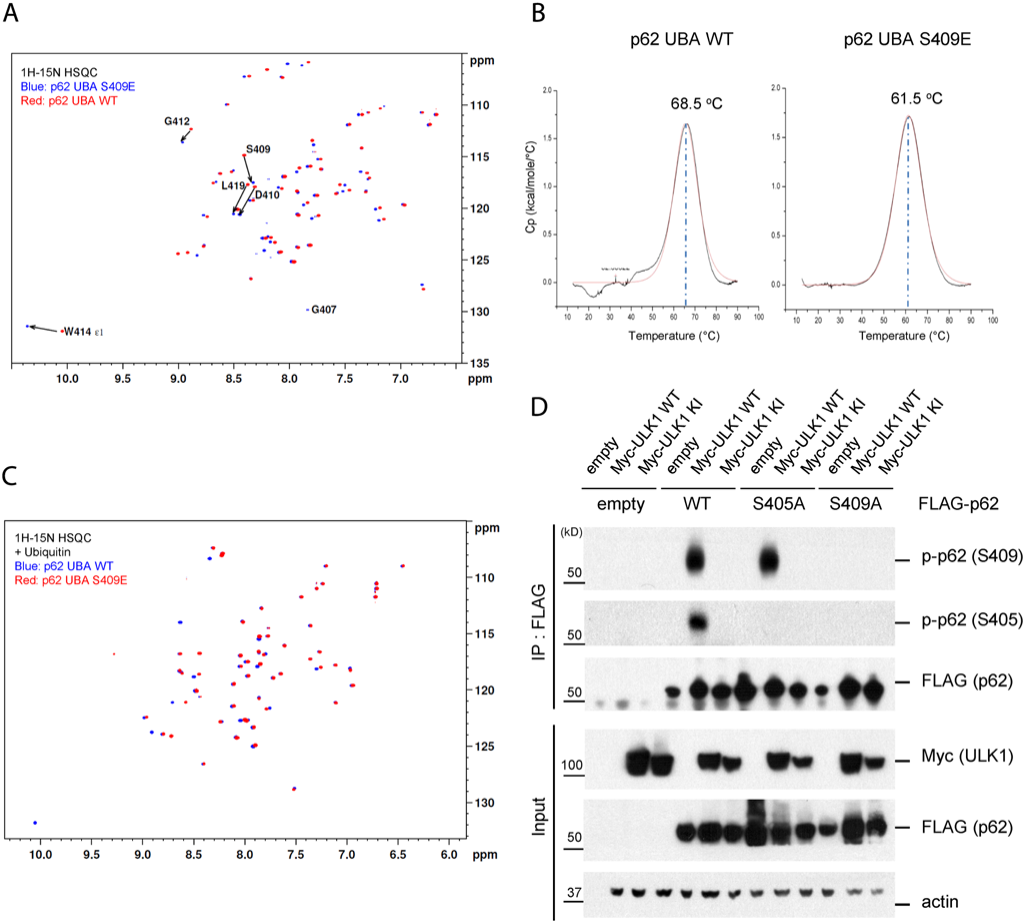
Phosphorylation of S409 destabilizes the UBA dimer interface and regulates phosphorylation of S405. **A**. S409E does not influence overall folding of UBA. Overlay of ^1^H-^15^N HSQC spectra of ^15^N-labeled p62 UBA WT(red) and S409E(blue) in the absence of mono-ubiquitin(Ub). **B**. p62 UBA S409E destabilizes p62 UBA dimer. Differential Scanning Calorimetry was performed with p62 UBA WT and S409E to measure the melting temperature of p62 UBA dimer. **C**. Overlay of ^1^H-^15^N HSQC spectra of ^15^N-labeled p62 UBA WT(blue) and S409E(red) in the presence of 6-fold excess unlabeled mono-Ub. **D**. ULK1 phosphorylates p62 at S405 and p-Ser409 is a prerequisite for ULK1-mediated p-S405. HEK 293T cells were transfected with indicated plasmids. IP with anti-FLAG antibody was performed and Westernblot assay with indicated antibodies was followed.

Previous structural analysis indicated that the p62 UBA domain dimerizes and exists in a dimer-monomer equilibrium in solution, while there is a shift of dimer to monomer of UBA domain upon binding to Ub [41,42]. To study the structural basis of how p-S409 influences Ub binding, we performed NMR spectrum analysis. The ^1^H-^15^N correlation spectra of the ^15^N-labeled p62 UBA WT and S409E were collected in the absence of ubiquitin (Fig. 6A). The overall dispersion patterns of cross peaks for S409E mutant and WT were similar. However, a few chemical shifts were significant. For example, the residues D410, G412, W414 and L418 were evidently altered in position using the published WT spectra as reference [41]. The “shift” residues are located in the vicinity of S409. Thus the S409E mutation altered these residues’ local environment and led to chemical shift in the HSQC spectra. Noticeably, residues W414 and L418 locate at the UBA dimer interface and are both important residues for dimer formation in the WT structure. The result suggests that the chemical perturbation by S409E leads to local destabilization of the UBA-UBA dimer interface. To test this idea, we examined overall thermal stability of the dimer conformation. We measured melting temperature(Tm) of p62 UBA WT and S409E by Differential Scanning Calorimetry(DSC). The UBA S409E mutant showed a much lower Tm(61.5°C) than WT(68.5°C) (Fig. 6B), supporting the hypothesis that the S409E UBA forms a less-stable dimeric structure than the p62 WT UBA.

Furthermore, we carried out NMR titration experiments by adding 6-fold molar excess of unlabeled mono-Ub to the p62 UBA WT and S409E. The addition of mono-Ub induced a large set of chemical shifts in the spectra of S409E, consistent with the idea that S409E UBA undergoes the dimer-monomer transition upon Ub binding as reported in the previous studies for WT p62 UBA domain [41]. The analysis, however, revealed that the disperse pattern of S409E UBA in the presence of Ub is similar to that of WT, with only a few noticeable chemical shifts involving residues such as S409E and W414 (Fig. 6C). These data suggest that S409E mutant follows the similar pattern as the WT in the overall folding of UBA structures in the absence or presence of Ub. Collectively, our data suggest that the S409E mutant destabilizes the UBA dimeric structure without impacting overall folding of the UBA domain bound to ubiquitin.

Previous reports showed that S405 of p62(equivalent to human S403) can be phosphorylated by different kinases(i.e. CK2, TBK-1 and ULK1) [27,28,35]. Consistent with a recent report [35], we also observed ULK1-dependent S405 phosphorylation in response to ULK1 WT overexpression, but not ULK1 KI mutant (Fig. 6D). Interestingly, mutation of S409A precluded p62 phosphorylation at S405, suggesting that S405 phosphorylation depends on S409 phosphorylation. Furthermore, we observed that S405A mutation did not affect ULK1-dependent phosphorylation of p62 at S409. Thus it is likely that p-S409 precedes p-S405 or that p-S409 is required for the stability of p-S405.

### p62 phosphorylation at S409 is necessary for autophagic degradation of polyubiquitinated proteins and recruiting autophagy machinery proteins

We next investigated how phosphorylation of S409 in p62 affects the autophagic degradation of poly-Ub proteins. We first examined p62 and ubiquitin co-localization upon MG132 and under normal culture condition in MEFs stably expressing FLAG-p62 variants. IF staining pattern of p62 WT, S409A and S409E appeared similar under normal culture conditions (S5 Fig., left). However, upon MG132 treatment, cells expressing the S409A mutant formed large protein aggregates that are labeled with Ub antibody, in contrast to the distribution of p62 in small and dispersed puncta observed in WT or S409E MEFs. These results suggested a likely block in the degradation of p62 and ubiquitinated proteins when p62 cannot be phosphorylated at S409 (S5 Fig., right).

**Fig 7:**
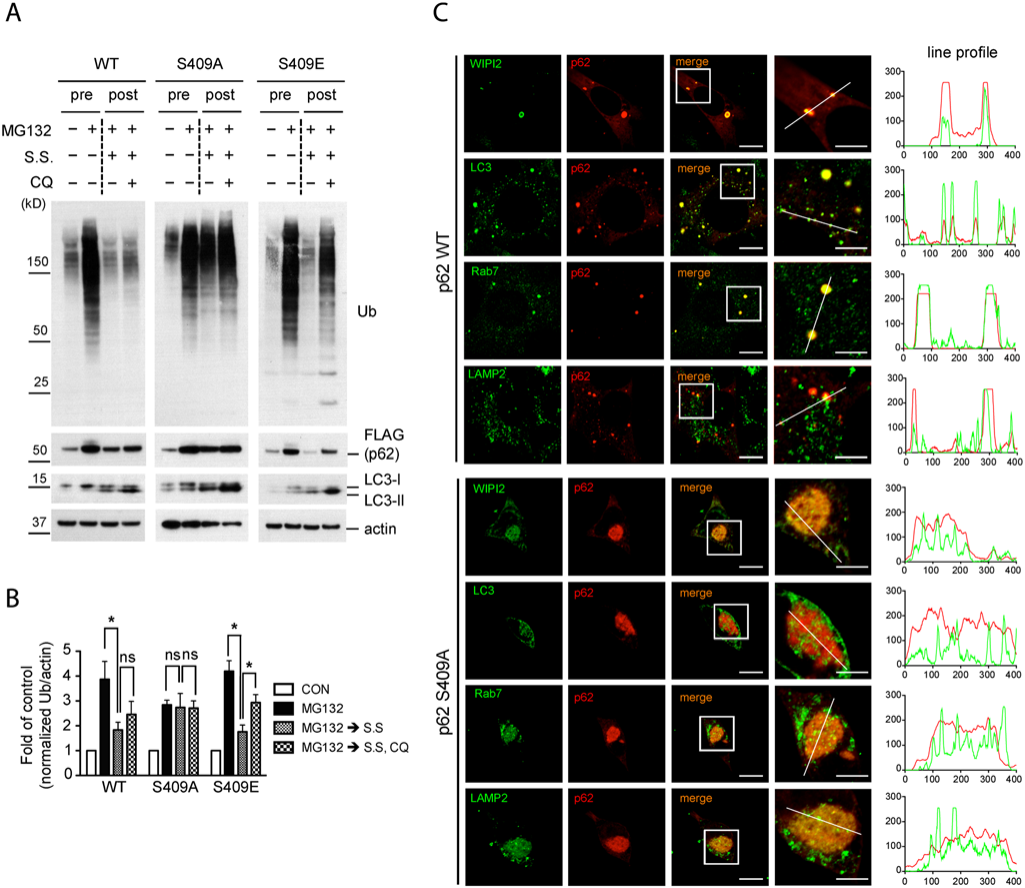
Phosphorylation of p62 at S409 is required for autophagic degradation of polyubiquitinated proteins and the recruitment of autophagy proteins. **A.-C**. p62 p-S409 regulates autophagic degradation of ubiquitinated proteins. p62 KO MEFs stably expressing indicated constructs were treated with MG132 for 16 hr(pre), then media were switched to serum starvation(S.S.), in combination with CQ for 24 hr(post). **A**. p62 S409A overexpressing cells are resistant to autophagic degradation of poly-Ub proteins. Total cellular lysates were subjected to immunoblot assay with indicated antibodies. **B**. The level of poly-Ub proteins from Fig. 7A was normalized to actin level, and further to each own control. Student’s *t*-test was used and data are represented as mean ± SEM(n = 4). * *p* < 0.05; ns, not significant **C**. p62 S409A is impaired in the recruitment of autophagy machinery proteins. MEFs were fixed, stained with antibodies against p62(red) and WIPI2, LC3, Rab7, or LAMP2(green), and then visualized under fluorescent microscope. Line profile was used to illustrate co-localization. Green lines indicate WIPI2, LC3, Rab7, or LAMP2 and red lines indicate p62. Scale bar = 10 μm, 5 μm for enlarged images.

To test the above idea, we treated MEFs stably expressing FLAG-p62 variants with MG132(pre) and then with serum starvation(S.S.) to induce autophagic degradation of accumulated poly-Ub proteins(post) [43]. Serum starvation efficiently cleared the accumulated poly-Ub proteins that follow MG132 treatment in both p62 WT and S409E MEFs. Interestingly, chloroquine(CQ, a blocker of lysosomal degradation) treatment results in significant accumulation of ubiquitinated proteins in p62 S409E, suggesting that S409 phosphorylation of p62 mediates the degradation of ubiquitinated proteins mainly through autophagy (Figs. 7A, B). In contrast, accumulation of poly-Ub proteins remained mostly unaltered with serum starvation conditions in S409A MEFs, and further CQ treatment had little effect in the amount of poly-Ub proteins compared to WT or S409E MEFs, suggesting a block in the autophagic degradation of poly-Ub proteins in S409A MEFs (Figs. 7A, B).

We next evaluated a number of autophagy markers in the context of p62-associated aggregates by using imaging analysis. IF staining results showed that p62 WT puncta co-localized with autophagy-related proteins, such as WIPI2, LC3, Rab7 and LAMP2 in small and dispersed dots, whereas p62 S409A formed large inclusions, which randomly sequester cellular proteins including some autophagy-related proteins, as a fraction of the autophagy proteins were seen outside of the inclusions (Fig. 7C). These results strongly suggest that p62 phosphorylation at S409 is critical for linking aggregated poly-Ub proteins to autophagy compartments for their efficient degradation.

### Phosphorylation of p62 at S409 potentiates autophagic degradation of polyQ-expanded Htt mutant

**Fig 8:**
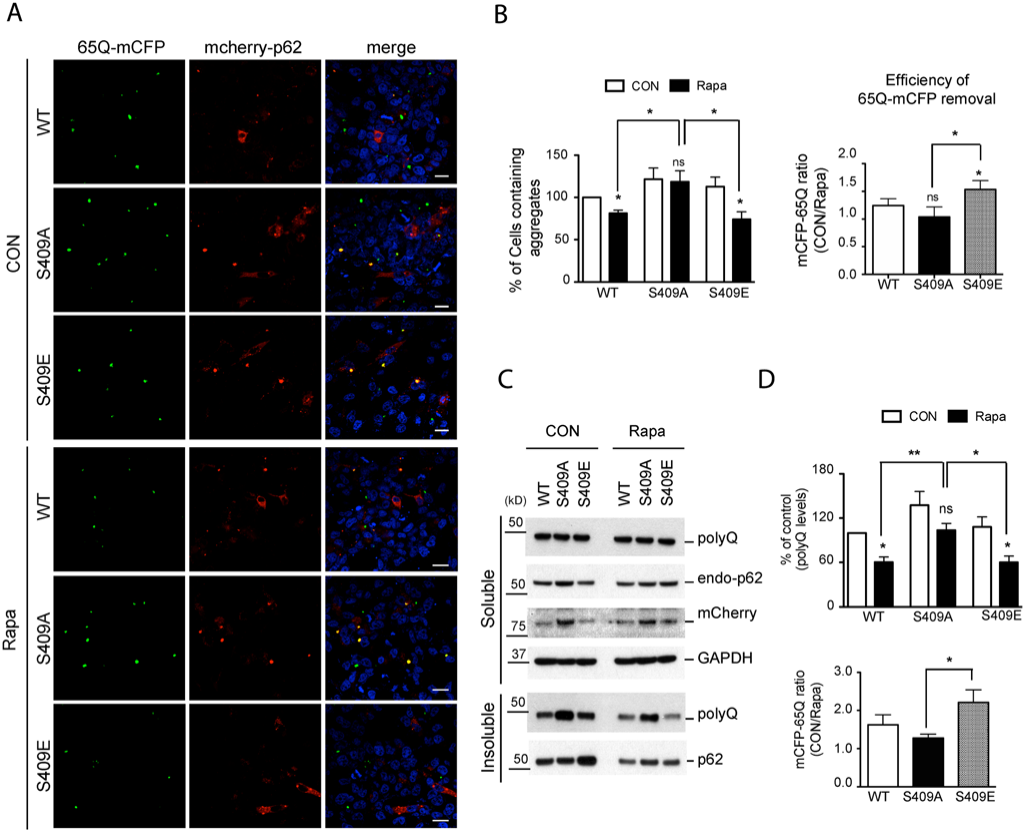
Phosphorylation of p62 at S409 enhances the autophagic degradation of polyQ-Htt mutant proteins. HeLa/65Q–mCFP cells were transfected with mCherry-p62 WT, S409A or S409E and treated with Rapamycin to induce autophagy. **A**. Cells were fixed and visualized under fluorescent confocal microscope. Scale bar = 20 μm. **B**. Quantifications of the results in Fig. 8A were performed by counting cell numbers containing 65Q-mCFP aggregates. Then the number of cell carrying 65Q-mCFP aggregates was normalized to the number of control cells transfected with p62 WT(left panel). Efficiency of polyQ clearance(right panel) was obtained from the ratio of control sample vs. rapamycin-treated sample. **C**. Cells were separated into detergent soluble and insoluble fractions and probed with indicated antibodies. **D**. Quantifications were performed by normalizing the intensity to that of control samples transfected with p62 WT(upper panel). Efficiency of polyQ reduction(lower panel) based on the results in Fig. 8C was obtained as described in Fig. 8B. One sample *t*-test and student *t*-test were used and data are represented as mean ± SEM(n = 4). * *p* < 0.05, ** *p* < 0.01; ns, not significant.

Finally, to test the role of p-S409 in degradation of disease protein aggregates, we overexpressed the mCherry-p62 variants in HeLa/65Q-mCFP. Induction of 65Q-mCFP expression in cells transfected with p62 WT or S409E resulted in a decrease in the number of cells positive for 65Q-mCFP aggregates when autophagy was enhanced with rapamycin treatment, as compared to normal culture conditions. In contrast, in S409A overexpressing cells, rapamycin did not affect the number of cells producing 65Q-mCFP aggregates (Figs. 8A, B). While the cell number containing 65Q-mCFP aggregates were significantly lower in p62 WT- or S409E- transfected cells than in S409A-transfected cells with rapamycin treatment, the efficiency for rapamycin-stimulated clearance of 65Q-mCFP aggregates was the highest in S409E cells compared to WT or S409A cells, based on the ratio of cell numbers with 65Q-mCFP aggregates in the absence or presence of rapamycin (Fig. 8B).

We then performed immunoblot analysis of 65Q-mCFP levels in those same transfected HeLa cells to confirm the effects of mCherry-p62 variants. We found that the majority of 65Q-mCFP remained in a detergent-insoluble fraction and that incubation with rapamycin had little effect on 65Q-mCFP levels in the soluble fraction in all cases. However, rapamycin treatment resulted in a reduction in the levels of insoluble 65Q-mCFP protein in p62 WT- or S409E- expressing cells, but not S409A-expressing cells (Figs. 8C, D). Indeed, 65Q-mCFP levels were significantly higher in S409A cells than WT or S409E cells after rapamycin treatment. Accordingly, the efficiency of autophagy-stimulated clearance(ratio of 65Q-mCFP levels before *versus* after rapamycin treatment) of S409E was higher than that of WT or S409A mutant (Fig. 8C). These results suggested that phosphorylation of p62 at S409 potentiates autophagic degradation of 65Q-mCFP.

## Discussion

**Fig 9:**
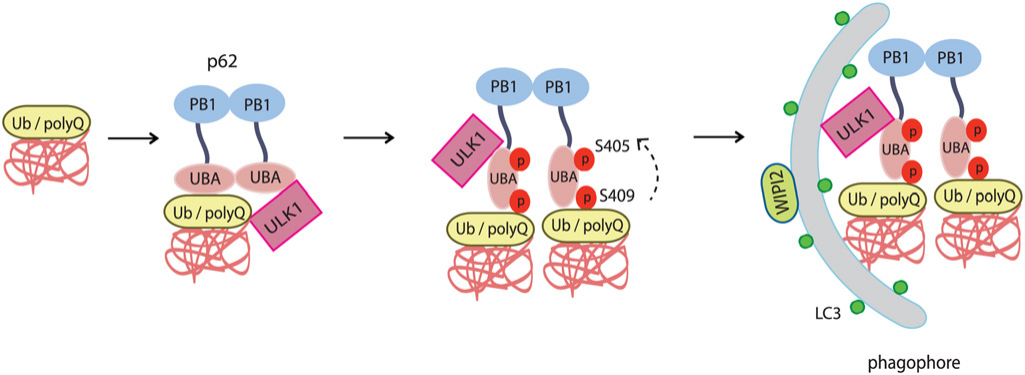
The working model for ULK1-mediated p-S409 and p-S405 of p62 in selective degradation of ubiquitinated proteins and polyglutamine-expanded proteins. Accumulated polyubiquitinated(poly-Ub) proteins or polyQ-expanded proteins trigger interaction of p62 with ULK1. This interaction induces ULK1-mediated p62 phosphorylation at S409 in UBA domain, which facilitates dimer to monomer transition of UBA domain, and subsequent phosphorylation at S405(mediated by either ULK1, CK2 or TBK-1). As a result, the phosphorylation of p62 at S405 and S409 leads to enhanced binding affinity of p62 to poly-Ub or polyQ-expanded proteins. The presence of ULK1 and p62 p-S405 and p-S409 in poly-Ub or polyQ-expanded protein aggregates causes the recruitment of autophagy machinery that is responsible for the degradation of poly-Ub or polyQ-Htt mutant proteins.

Our study reveals that proteotoxic conditions trigger a response of selective autophagy involving phosphorylation of autophagy receptor p62/SQSTM1 by ULK1. ULK1-mediated phosphorylation of S409 as well as S405 in its UBA domain occurs in response to ubiquitinated protein accumulation(upon proteasome inhibition) and aggregate-prone polyQ-expanded Htt protein that is causal to Huntington’s disease(see our working model in Fig. 9). In contrast, p62 S409 phosphorylation does not occur when cells are starved of amino acid or glucose, conditions upon which ULK1 is typically activated to induce macroautophagy, a bulk degradation pathway with little selectivity of substrates [6,9]. Thus our study suggests that the ULK1-p62 cascade plays an important role in the regulation of selective autophagy. The increased binding of modified p62 with ubiquitinated cargo supports the idea that the signaling cascade responds to pathological protein aggregates by increasing their recognition and degradation. The lack of co-localization between the non-phosphorylated p62(S409A) mutant and autophagy markers suggests that p62 S409A fails to recognize autophagic cargos and recruit the autophagy machinery.

Our data indicates that ULK1-mediated phosphorylation of p62 is likely activated by mechanisms distinct from canonical nutrient pathways (Fig. 2C). Instead, it may involve sensing of proteotoxic stresses such as accumulation of misfolded poly-ubiquitinated proteins (Fig. 2) or disease-related protein aggregates (Fig. 3). The exact molecular mechanism by which upstream signaling activates the ULK1-p62 cascade remains to be characterized. Our data suggest that accumulation of protein aggregates triggers the interaction of ULK1 with p62 (Fig. 4) and subsequent ULK1-dependent phosphorylation of p62 at two serine sites in p62’s UBA domain. Consistently, ULK1-p62 interaction involves ULK1’s kinase domain and the ability of p62’s UBA domain to bind Ub (S3 Fig.). Our results also suggest that S409 phosphorylation, similar to that of S405, is important for linking autophagic cargos to the autophagy machinery, degradation of ubiquitinated proteins and polyQ-expanded proteins (Figs. 6, 7). To our surprise, a recent report showed that deletion of *p62* ameliorated the pathology in R6/2 HD mouse model [44], in contrast to the previous evidence that *p62* depletion exacerbated disease progression in spinal and bulbar muscular atrophy(SBMA), another polyQ-associated disease [45]. Given that p62 is a multifaceted protein that is involved in multiple cellular pathways [46] and that it shuttles between the nucleus and cytoplasm [47], the toxicity of p62 as described in the report could be related to nuclear shuttling of mutant huntingtin or/and the de-regulation of Keap1-Nrf2 pathway in R6/2 HD mice. Revelation of the underlying mechanisms that determine the outcome of *p62* deletion in different disease models will aid in understanding the pathophysiology of each disease and developing specific therapeutic targets.

Our study also provides novel structural and functional insights into the role of the novel S409 and previously identified S405 p62 phosphorylation in regulating ubiquitinated protein binding [27,28,35]. Previous studies showed that the UBA of p62 has only weak affinity to free Ub [40] or unanchored tetra-Ub [48], casting a doubt on the significance of p62’s UBA domain in ubiquitinated cargo binding. Here our study demonstrates that the increased affinity of p62 UBA to ubiquitinated proteins is achieved following ULK1-dependent phosphorylation of p62 at S409 and S405. The p62 UBA domain exists in dimer–monomer equilibrium *in vitro*, with the dimer conformation incompatible for interaction with Ub [41,42]. Modulation of the dimer—monomer transition of UBA domain likely plays a role in regulating p62 function in the recognition and degradation of ubiquitinated proteins, although no experimental evidence had been reported previously. Our NMR analysis also shows that, although the overall structural folding of the p62 UBA S409E differs little from that of WT p62(either before or after Ub binding), the S409E mutation alters the local relationship between select critical residues, including W414 and L418 at the p62 UBA-UBA dimer interface (Figs. 6A, C) [41]. Furthermore the overall thermal stability of the UBA domain is significantly reduced in the S409E mutant, suggesting a reduced interaction between the two S409E monomers (Fig. 6B). Thus we conclude that phosphorylation at S409 causes a destabilized dimer interface and facilitates dimer–monomer transition in favor of binding to ubiquitinated proteins. This event could also be connected to the requirement of S409 phosphorylation for the subsequent S405 phosphorylation, while p-S405 is dispensable for p-S409. In fact, the S405 and S409 are separated by 3 amino acids,(MGF), a conserved motif on the L1 loop of the UBA domain important for Ub binding [41]. While S409 is located at the L1 loop of the dimer interface, S405 resides at the end of a α-helix required for Ub binding. We thus propose a model in which S409 is phosphorylated first by ULK1 to promote a dimer to monomer transition, followed by the exposure of S405 to ULK1(or CK2 or TBK-1) for further phosphorylation (Fig. 6D). In this model, and based on our observations, p62 phosphorylation at S405 and S409 might have distinct roles in modulating p62-Ub binding: while located at the Ub binding site, p-S405 results in enhanced affinity between Ub and p62 perhaps via charged residue interactions; in contrast, p-S409 destabilizes the UBA dimer interface and allows S405 phosphorylation to occur or maintains the steady state of p-S405, potentiating Ub and p62 interaction.

Although the crosstalk between autophagy and ubiquitin proteasome system(UPS) has been described previously and available evidence suggests that autophagy can be activated as a salvage pathway under UPS impairment [3,49,50], the exact molecular mechanism is not well understood. Undigested proteasomal substrates resulting from a block of the UPS become sequestered in Ub-positive protein inclusions also known as aggresomes [51] or p62 bodies [52], where the autophagy receptor p62 tethers ubiquitinated cargos to the autophagy machinery via LC3 for degradation. Thus our study provides insights into the mechanism for the cross-talk between the UPS and the autophagy pathway whereby enhanced autophagy, through ULK1-p62 coordinated action, compensates for the inhibition of UPS degradation to clear ubiquitinated proteins. It remains to be shown whether other types of ubiquitinated cargos, such as injured mitochondria, peroxisomes and invading microbes can also be recognized and degraded through the similar ULK1-p62 mechanism.

In summary, our study reveals a molecular and structural mechanism underlying the autophagy receptor p62-mediated degradation of ubiquitinated or aggregated disease proteins through selective autophagy. Our results thus provide a rationale for the development of therapeutics against human diseases associated with protein aggregates(proteinopathies), based on ULK1 and p62 interaction and signaling.

## Materials and Methods

### Ethics statement

All animal studies were performed in compliance with IACUC(Institutional Animal Care and Use Committee) at Icahn School of Medicine at Mount Sinai.

### Reagents

MG132(calbiochem), chloroquine(CQ; Sigma-Aldrich), polybrene(Sigma-Aldrich), lipofectamine 2000(Invitrogen), puromycin(InvivoGen), EDTA-free protease inhibitor cocktail and phosphatase inhibitor cocktail(Roche Diagnostics), mouse monoclonal M2 FLAG affinity gel beads(Sigma-Aldrich), ammonium bicarbonate(NH_4_HCO_3_; Sigma-Aldrich), iodoacetamide(IAM; Sigma-Aldrich), formic acid(Sigma-Aldrich), Trifluoroacetic acid(TFA; Pierce), tris(2-carboxyethyl)phosphine(TCEP; Pierce), bovine trypsin(Roche Applied Science), acetonitrile(ACN;Thermo Fisher Scientific), POROS 20 R2 beads(Applied Biosystems), C18 ZipTips(Merck Millipore), isopropyl-β-D-thiogalactopyranoside(IPTG; Sigma-Aldrich), Dynabeads protein G(Invitrogen), protein G Sepharose(GE Healthcare Life Sciences), NuPAGE Bis-Tris and Tris-Acetate gels running system(Invitrogen), QuikChange Lightning Site-Directed Mutagenesis Kit(Agilent Technologies), Hybond-P PVDF membrane(GE Healthcare Life Sciences), BCA Protein Assay Reagent Kit(Pierce), Radioactive [γ-^32^P]ATP(PerkinElmer), Calf intestinal alkaline phosphatase(New England Biolabs), Factor Xa(New England Biolabs), K48- and K63-linked poly ubiquitin chains(Boston Biochemical), DAPI-containing fluorescence mounting medium(Invitrogen) were purchased from indicated suppliers.

### Antibodies

ULK1(Sigma-Aldrich, #A7481), p62(Progen Biotechnik, #GP62-C), p-p62 Ser403(Millipore, #MABC186), β-Actin(Cell Signaling Technology, #3700), FLAG-M2(Sigma-Aldrich, #F1804), myc(Cell Signaling Technology, 9B11, #2276), Ubiquitin(Dako, #Z0458; Abcam, #ab7780; Biomol, clone FK2, #PW8810), LC3B(Cell signaling, #2775), WIPI2(abcam, #ab101985), Rab7(Cell signaling, #9367), LAMP2(DSHB, #H4B4), GAPDH(Chemicon, #MAB374), polyQ(Merck Millipore, #MAB1574), GFP(Life technologies, #A11122), DsRed(Clontech lab, #632496) were purchased from the indicated suppliers. Anti–phosphorylated p62 polyclonal antibody was raised in rabbits using the peptide SMGF(pS)DEGGWLTRC as an antigen by Abgent and Cocalico Biologicals.

### Plasmid constructs

FLAG p62-wild type,-F408V,-ΔPB1, and MBP p62-wild type,-M1,-M4,-M7 constructs were provided by Dr. Masaaki Komatsu(Niigata University). Myc-ULK1 wild type and kinase inactivated(K46I) mutant were provided by Dr. Sharon Tooze(London Research Institute). FLAG ULK1-wild type,-Δkinase,-ΔC,-ΔS/ΔC constructs were provided by Dr. Mondira Kundu(St. Jude Children’s Research Hospital). LPC retroviral vector and helper vector were provided by Dr. Wei-Xing Zong(Stony Brook University). FLAG-p62 was cloned into HindIII and XhoI sites of LPC retroviral vector. mCherry p62-wild type was provided by Dr. Thomas Weber(Icahn School of Medicine at Mount Sinai). The full length of mouse ubiquitin was inserted into the modified pET32 vector between the restriction sites BamHI and EcoRI as a Trx-His_6_-tagged protein. The UBA domain of mouse p62(residues 391–438) was cloned as a GST-His_6_-tagged protein in a modified pET49 vector containing the human rhinovirus(HRV) 3C protease cleavage site. Based on each p62 wild type constructs, site-directed point mutation was performed to substitute serine 409 to either alanine(S409A) or glutamic acid(S409E) and serine 405 to alanine(S405A).

### Cell cultures

HEK 293T cells and MEFs were maintained in Dulbecco’s modified Eagle’s medium(DMEM, Invitrogen) supplemented with 10% fetal bovine serum(FBS, Invitrogen) and 50 μg/ml penicillin and streptomycin(Invitrogen). Immortalized wild type and p62 knockout Mouse Embryonic Fibroblasts(MEFs) are provided by Dr. Masaaki Komatsu(Niigata University) [53] and Dr. Mondira Kundu(St. Jude Children’s Research Hospital) provided wild type, ULK1 knockout, and ULK1/2 double knockout MEFs [38]. HeLa/polyQ-mCFP cells were maintained as previously described [33], which is generous gifts from Dr. Ai Yamamoto(Columbia University). Transient transfection was performed using Lipofectamine 2000 as per the manufacturer’s instruction.

### Retroviral infection and generation of stable cell lines

Retroviral infection was done as described previously [54]. Briefly, HEK 293T cells were transfected with LPC-retroviral constructs and helper viral construct. After 24 hr, the supernatant was collected and filtered through 0.45 μm pore size nylon filter and supplemented with 10 μg/ml of Polybrene. p62 KO MEFs were infected with this supernatant and then selected with 3 μg/ml of Puromycin. Single colonies were picked and cultured.

### Immunoblot analysis

For total cellular lysates, cells were lysed on ice in TNTE buffer(1% Triton X-100, 20 mM Tris-HCl pH 7.5, 120 mM NaCl, 1 mM EDTA) containing 1% SDS, complete protease inhibitor cocktail, and phosphatase inhibitor cocktail. Homogenization using a 1 ml syringe with a 26-gauge needle was followed and supernatants were collected after centrifugation at 13,000 g for 15 min at 4°C. Supernatants were subjected to BCA assay and then were resolved by SDS-PAGE. For immunoprecipitation, cells were lysed in RIPA buffer(50 mM Tris-HCl pH 7.4, 1% NP-40, 0.25% Na-deoxycholate, 150 mM NaCl, 1 mM EDTA) containing complete protease inhibitor cocktail, and phosphatase inhibitor cocktail for 30 min at 4°C. After centrifugation at 13,000 g for 15 min at 4°C, collected supernatants were incubated with antibodies overnight at 4°C. Lysates were further incubated with Dynabeads protein G or protein G Sepharose for 1.5 hr at 4°C and then washed with RIPA buffer 5 times and subjected to immunoblot assay.

### Fluorescence microscopy

Cells were fixed in 4% paraformaldehyde in PBS for 30 min and permeabilized in PBS containing 0.1% saponin for 10 min at room temperature. Cells were further blocked in PBS containing 5% goat serum and 0.2% Triton X-100 for 1 hr and then incubated with primary antibodies in PBS containing 1% goat serum and 0.2% Triton X-100 overnight at 4°C. For phospho-p62(S409) antibody, PBS containing 0.2% Triton X-100 and 3% BSA or 1% BSA for blocking and antibody incubation, respectively, was used. After washing with PBS three times, cells were incubated with Alexa-conjugated secondary antibody for 1 hr at room temperature. Secondary antibodies used are goat anti-rabbit Alexa Fluor 555 and 488, goat anti-rat Alexa Fluor 488, goat anti-mouse Alexa Fluor 488, goat anti-guinea pig Alexa Fluor 555 and 647. Washing with PBS was followed and then mounted with mounting medium(ProLong Gold antifade mountant with DAPI, Invitrogen). Cells were examined under Carl Zeiss upright or invert confocal microscopes(LSM780 system). Images were taken with 63X oil immersion objective lens at room temperature and image acquisition was performed by Zen2012 software. Digitized images were analyzed and processed by using Image J software. Line profiling was performed with Image J software.

### Preparation of brain lysates

All animal studies were performed in compliance with IACUC(Institutional Animal Care and Use Committee) at Icahn School of Medicine at Mount Sinai. Floxed *Atg7* mice(*Atg7* ^f/f^) were characterized previously and were crossed with *Synapsin-Cre* mice to generate *Atg7* ^f/f^:*Synapsin-Cre* mice [1]. Whole brains of *Atg7* ^f/f^: *p62* ^+/ −^, *Atg7* ^f/f^: *nestin-Cre*: *p62* ^+/ −^, *Atg7* ^f/f^: *nestin-Cre*: *p62* ^−/−^, *Atg7* ^f/+^: *nestin-Cre*: *p62* ^−/ −^ were provided by Dr. Masaaki Komatsu. Brain lysates of mice brains were prepared as described previously [22]. z-Q175 KI line was derived from the CAG 140 KI mouse model and carries around 175 CAG repeats [36]. Heterozygous z_Q175 and its age-matched wild type littermate control were obtained from the CHDI colony at the Jackson Laboratories. Cortical and Striatal lysates were prepared with RIPA buffer supplemented with 1% SDS, complete protease inhibitor cocktail, and phosphatase inhibitor cocktail, followed by homogenizing tissues with blue pestles and heating at 60°C for 1 hr. Supernatants obtained after centrifugation at 15, 000 g for 30 min at 4°C were subjected to immunoblot assay.

### Triton X-100-soluble and insoluble fractionation

Cells were lysed on ice 1% Triton X-100 in PBS supplemented with complete protease and phosphatase inhibitor cocktails for 30 min. After centrifugation at 15,000 g for 30 min at 4°C, 1% Triton X-100-soluble fractions were collected. The pellets were washed four times with 1% Triton X-100 in PBS and further solubilized with addition of 1% SDS for 1 hr at 60°C. Subsequently, Triton X-100-insoluble fractions were collected by centrifugation at 15,000 g for 30 min at 4°C and protein samples were submitted to immunoblot assay.

### Protein expression and purification

Expression of MBP-p62 WT and mutants were induced in *E.Coli* BL21(DE3) cells by growing at 25°C for 16 hr with 0.05 mM of isopropyl-β-D-thiogalactopyranoside(IPTG). Bacterial cells were lysed with TNE buffer(10 mM Tris-HCl pH 8.0, 150 mM NaCl, 1 mM EDTA, and 1% NP-40) and centrifuged for 20 min at 9,000 g at 4°C. Supernatants were incubated with amylose resin at 4°C for overnight and MBP-p62 bound resins were washed three times with TNE buffer. Subsequently, MBP-p62 proteins were eluted by 10 mM of maltose in 20 mM Tris-HCl pH 7.5, 150 mM NaCl. GST-His_6_ Ubiquitin full length and UBA domain constructs were expressed in *E*. *coli* BL21(DE3) cells at 30°C after induction by IPTG and purified by affinity chromatography(HisTrap HP, GE Healthcare). The Trx- or GST-His_6_ tags were removed by 3C cleavage and the untagged proteins were further purified by size-exclusion chromatography(Superdex 75, GE healthcare).

### *In vitro* phosphorylation and dephosphorylation assay

*In vitro* p62 phosphorylation assay by ULK1 was performed as described before [6]. In brief, MBP tag of purified MBP-p62 proteins was cleaved with Factor Xa and then incubated with Myc-ULK1 immunoprecipitants from transfected HEK 293T cells in Kinase buffer(20 mM HEPES at pH 7.4, 1 mM EGTA, 0.4 mM EDTA, 5 mM MgCl2 and 0.05 mM DTT(dithiothreitol) containing 100 μM of cold ATP and 5 μCi [γ-^32^P]-ATP per reaction at 37°C for 30 min. The reaction was terminated by adding SDS sample buffer and subjected to SDS–PAGE and autoradiography. For cold reaction, 500 μM of cold ATP was used instead and subjected to immunoblot assay with customized pS409-p62 antibody.

For dephosphorylation assay, the membrane incubated with p-p62 S409 antibody was further incubated with alkaline phosphatase for 1 hr at 37°C. SDS sample buffer was added to stop reaction and then SDS-PAGE and autoradiography or immunoblot assay with pS409-p62 antibody were performed.

### Ub binding assay

For cell-based poly-Ub binding assay, p62 KO MEFs stably transfected with FLAG-p62 WT, S409A, S409E, or empty vector were lysed with RIPA buffer. To generate poly-Ub proteins, normal p62 KO MEFs were treated with MG132 and then lysed with RIPA buffer. Same amount of protein lysates from each pool were mixed and then incubated with M2 FLAG affinity gel beads for overnight at 4°C. Beads were extensively washed with RIPA buffer five times and then subjected to immunoblot assay.

For the *in vitro* linkage specific Ub binding assay, 15 μg of purified MBP-p62 wild type and mutant proteins were incubated with amylose resin in reaction buffer(50mM HEPES, pH 7.5, 10% Glycerol, 150mM NaCl, 1% Triton X-100, 1mM EDTA, 1mM EGTA) at 4°C for 1 hr. Incubation with 0.8 μg of K48− or 0.4 μg of K63−linked poly Ub chains was followed at 4°C for 2 hr. Reactants were extensively washed five times with reaction buffer and then subjected to immunoblot assay.

### Isothermal Titration Calorimetry(ITC)

Isothermal Titration Calorimetry was performed using an iTC_200_ microcalorimeter(MicroCal Inc.). Samples were dialyzed into 50 mM Tris, pH 8.0, and 150 mM NaCl. For UBA-ubiquitin interactions the injection syringe was loaded with 40 μl of ubiquitin sample and the cell was loaded with 220 μl of the respective UBA sample including wild type and S409E. Typically titrations consisted of 20 injections of 2 μl, with 200-s equilibration between injections. The data were analyzed using Origin 7.0.

### NMR

All the ^1^H-^15^N HSQC spectra of WT and S409E p62 UBA domain were collected at a concentration of 100 μM protein in 20mM sodium phosphate buffer, pH6.8, 5mM potassium chloride, 1mM EDTA and 10% D_2_O. For the ubiquitin titration, 6-equimolar ubiquitin was mixed with the ^15^N labeled UBA samples before the data collection. The spectra were acquired with a Bruker Avance 700 MHz spectrometer at 20°C and data were processed by the software provided by the manufacturer(Bruker Corporation).

### Differential Scanning Calorimetry

Differential Scanning Calorimetry measurements were carried out using a MicroCal VP-DSC calorimeter(MicroCal Inc.) with 0.5ml cells under a constant pressure of 2.5 atm. For the thermal stability data collection, all the protein samples were exchanged to buffer containing 20 mM HEPES, pH7.4, 115 mM NaCl, 1.2 mM CaCl_2_, 1.2 mM MgCl_2_ and 2.4 mM K_2_HPO_4_ by dialysis. Five rounds of buffer to buffer scans from 10–90°C by a ratio of 60°C /hr were performed to acquire a high quality baseline and a consistent thermal history prior to the protein data collection. The protein samples at a concentration of 200 μM were degassed and warmed to 25°C before being loaded to the sample cell. All the protein samples were injected in a temperature window between 15–25°C when the cell cooling down to 10°C after the previous scan. Data were analyzed by the software provided by the manufacturer(MicroCal Inc.), including baseline subtraction, normalization and model fitting. For each experiment, at least three independent scans were performed.

### Mass spectrometric analysis

Mass spectrometric analysis to identify ULK1-mediated p62 phosphorylation was performed as described previously with slight modification [55]. HEK 293T cells transfected with FLAG-p62 along with either ULK1 wild type or kinase mutant were immunoprecipitated with anti-FLAG antibody and resolved by SDS-PAGE. The gel was stained with Coomassie blue and then p62 band was excised, followed by de-staining with 45% acetonitrile in 100 mM ammonium bicarbonate. Subsequently, gel slices were reduced with 10 mM tris(2-carboxyethyl) phosphine hydrochloride(TCEP) and alkylated with 50 mM iodoacetamide. The proteins in each gel piece were then subjected to trypsin digestion and the reactions were stopped by 5% formic acid in 0.2% TFA. The extracted peptides by using POROS 20 R2 beads were concentrated and desalted using C_18_ zip-tips and eluted with 0.1% TFA in 40% acetonitrile followed by 0.1% TFA in 80% acetonitrile. The eluents were dried down using a Speed Vac-concentrator and reconstituted with 0.1% formic acid in 2:98 ACN: H_2_O for liquid chromatography tandem mass spectrometry(LC-MS/MS) analysis. A NanoAcquity UPLC system(Waters) interfaced to an LTQ-Orbitrap mass spectrometer(Thermo Scientific) equipped with a nanospray ionization source was employed for LC/MS/MS analyses. Reversed-phase LC was performed on Waters BEH130 C_18_ column(100 μm x 100 mm, 1.7 μm particle size). Samples were trapped and washed in Waters Symmetry C_18_ trap column(180 μm x 100 mm, 5 μm particle size) prior to separation in the capillary column. Gradient elution was carried out with 0.1% formic acid in water as solvent A and in ACN as solvent B, with solvent B raised from 1 to 50% in 30 minutes, then 50 to 85% in the next 10 min. A flow rate of 0.5 μL/minute was used.

MS/MS spectra were searched against IPI mouse database(ver. 3.87) using Sequest(ver.27, Rev. 11), Mascot(Ver. 2.4.0, Matrix Science, UK) and X! Tandem(The GPM, thegpm.org; version CYCLONE(2010.12.01.1)) algorithms(1,2). Scaffold(version 4.2.1, Proteome Software Inc.,) was used to validate MS/MS based peptide and protein identifications. Searches were performed with full tryptic specificity(2 missed cleavages); carbamidomethylated cysteine residues as static modification; deamidated asparagine and glutamine(+0.9840 Da), oxidized methionine, histidine and tryptophan(+15.9949 Da), and phosphorylated serine, threonine and tyrosine(+79.9663 Da) as differential modifications. Scaffold PTM version 2.1.2.1(Proteome Software Inc., Portland, Oregon, USA) was used to annotate Post Translational Modification(PTM) sites contained in MS/MS spectra.

### Statistical analysis

All experiments were performed at least 3 times unless it is indicated. Data are presented as mean ± SEM from at least three independent experiments. Statistical analyses and graphing were performed with GraphPad Prism v5.0(GraphPad Software). One sample *t*-test and unpaired Student’s *t*-test were used. A *p* value less than 0.05 was considered as statistically significant.

## Supporting Information

S1 Fig ULK1 phosphorylates p62 at Ser409. **A**. Schematic diagrams of MBP-p62 WT and mutant constructs. MBP-p62 WT, M1(PB1 domain deletion mutant), M4(UBA domain deletion mutant) and M7(PB1 and UBA domain deletion mutant) are depicted. **B**. A gel image stained with coomassie blue. The concentrations of bacterially purified MBP-p62 variant proteins were decided by running and visualizing a gel along with BSA. Same amount of proteins were incubated with Factor Xa and then further subjected to *in vitro* ULK1 kinase assay for S1C Fig. **C**. UBA domain of p62 is a main target of ULK1-mediated phosphorylation. Purified MBP-p62 variant proteins were incubated with Myc-ULK1 WT or KI isolated from transfected HEK 293T cells. **D**. The annotated spectrum for the phosphopeptide 396–417 containing S409. Phosphorylation of p62 at S409 was identified by LC-MS/MS analysis.
(TIF)

S2 Fig The redundant role of ULK1 and ULK2 in p-S409 of p62 upon the accumulation of protein aggregates. **A**. ULK1 and ULK2 are responsible for p62 phosphorylation at Ser409 upon MG132 treatment. ULK WT and ULK1/2 double knockout(DKO) MEFs were transfected with empty vector or FLAG-p62 and treated with MG132 treatment. IP with FLAG antibody was performed and immunoprecipitants were analyzed with indicated antibodies. **B**. The accumulation of p62 p-S409 and p-S405 in autophagy deficient brain tissues. Whole brain lysates of *Atg7* ^f/f^ and *Atg7* ^f/f^; Synapsin-Cre were analyzed. **C**. Specificity of p-S409 in autophagy deficient brain tissues. Insoluble fraction from whole brain lysates of *Atg7* ^f/f^; *p62* ^+/-^, *Atg7* ^f/f^; nestin-Cre; *p62* ^+/-^, *Atg7* ^f/f^; nestin-Cre:*p62* ^-/-^, *Atg7* ^f/+^; nestin-Cre; *p62* ^-/-^ were immunoblotted with indicated antibodies. The gel was stained with Coomassie blue for loading control.
(TIF)

S3 Fig Interaction between p62 and ULK1 is mediated by ubiquitin binding activity of p62 and ULK1 kinase domain. **A**. ULK1-p62 interaction requires ubiquitin binding site of p62. p62 KO MEFs carrying empty vector, FLAG-p62 WT or F408V mutant were treated with MG132 and cellular lysates were subjected to IP with anti-ULK1 or-FLAG antibodies. Immunoprecipitants were detected with indicated antibodies. **B**. The level of IPed p62 or ubiquitinated proteins was initially normalized to the level of input and then further normalized to the level of IPed ULK1 or FLAG, respectively. One sample *t*-test was used and data are represented as mean ± SEM(n = 4). * *p* < 0.05, ** *p* < 0.01. **C**. ULK1 kinase activity is required for p62 binding. HEK 293T cells were transfected with empty vector, Myc-ULK1 WT or KI(kinase inactive mutant) together with FLAG-p62. IP with anti-Myc antibody was performed, followed by immunoblot assay. **D**. The kinase domain of ULK1 mediates p62 binding. A schematic diagram indicates ULK1 domain structures(Top). HEK 293T cells were transfected with mCherry-p62 WT along with FLAG-ULK1 WT, Δkinase(kinase deletion mutant), ΔC(CTD deletion mutant) or ΔS/ΔC(S/P spacer and CTD deletion mutant). Transfected cells were treated with MG132 and then used in IP with anti-FLAG antibody and analyzed with indicated antibodies. Asterisks indicate non-specific bands.
(TIF)

S4 Fig P-Ser409 of p62 enhances p62 and K63-Ub binding but not K48-Ub binding. **A**. P-Ser409 increases p62 and K63-Ub binding. Bacterially expressed MBP, MBP-p62 WT, S409A or S409E were subjected to pull down assay in the presence of K48-or K63-linked ubiquitin peptides. Interaction between Ub and p62 was detected by immunoblotting with Ub antibody(upper panel); MBP and MBP-p62 protein levels were confirmed by coomassie blue gel staining(lower panel). **B**. Ubiquitin levels pulled down by MBP-p62 S409A or S409E proteins were normalized to the protein input and compared to that of MBP-p62 WT protein. One sample *t*-test was used and data are represented as mean ± SEM(n = 4). * *p* < 0.05, ** *p* < 0.01
(TIF)

S5 Fig Subcellular localization of p62 variants and Ub upon MG132 treatment. p62 KO MEFs stably over-expressing p62 WT, S409A, or S409E were treated with MG132, fixed, stained with p62(green) and ubiquitin(red) antibodies, and then visualized under fluorescent microscopy. Scale bar = 10 μm.
(TIF)
