# Supplementary figures and images for: Proteotoxic Stress Induces Phosphorylation of p62/SQSTM1 by ULK1 to Regulate Selective Autophagic Clearance of Protein Aggregates
Source: PLoS Genet. 2015 Feb 27;11(2):e1004987. doi: 10.1371/journal.pgen.1004987 (PMC4344198; doi:10.1371/journal.pgen.1004987)

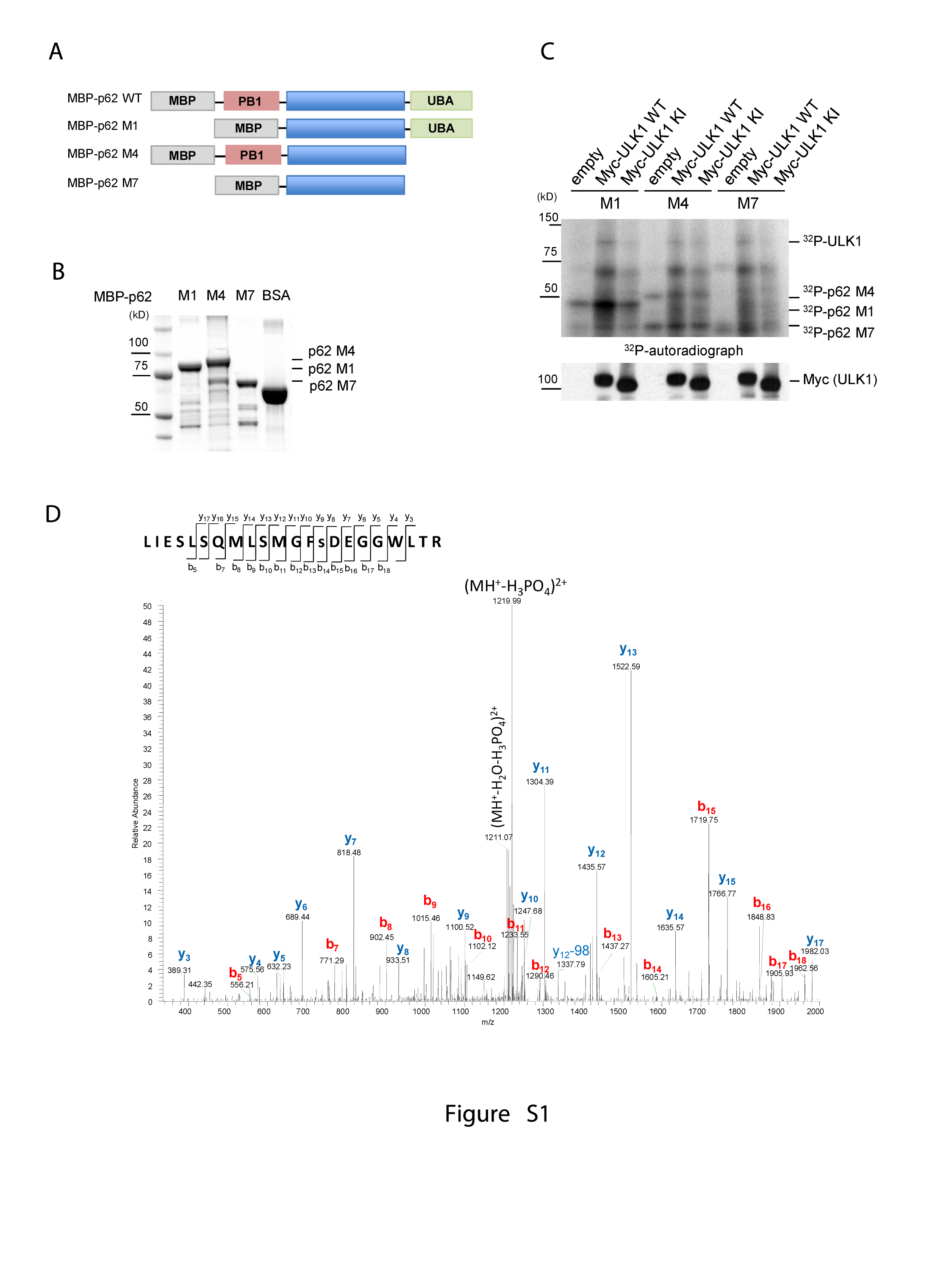

Supplement: S1 Fig — A. Schematic diagrams of MBP-p62 WT and mutant constructs. MBP-p62 WT, M1(PB1 domain deletion mutant), M4(UBA domain deletion mutant) and M7(PB1 and UBA domain deletion mutant) are depicted. B. A gel image stained with coomassie blue. The concentrations of bacterially purified MBP-p62 variant proteins were decided by running and visualizing a gel along with BSA. Same amount of proteins were incubated with Factor Xa and then further subjected to in vitro ULK1 kinase assay for S1C Fig. C. UBA domain of p62 is a main target of ULK1-mediated phosphorylation. Purified MBP-p62 variant proteins were incubated with Myc-ULK1 WT or KI isolated from transfected HEK 293T cells. D. The annotated spectrum for the phosphopeptide 396–417 containing S409. Phosphorylation of p62 at S409 was identified by LC-MS/MS analysis. (TIF) [file pgen.1004987.s001.tif]

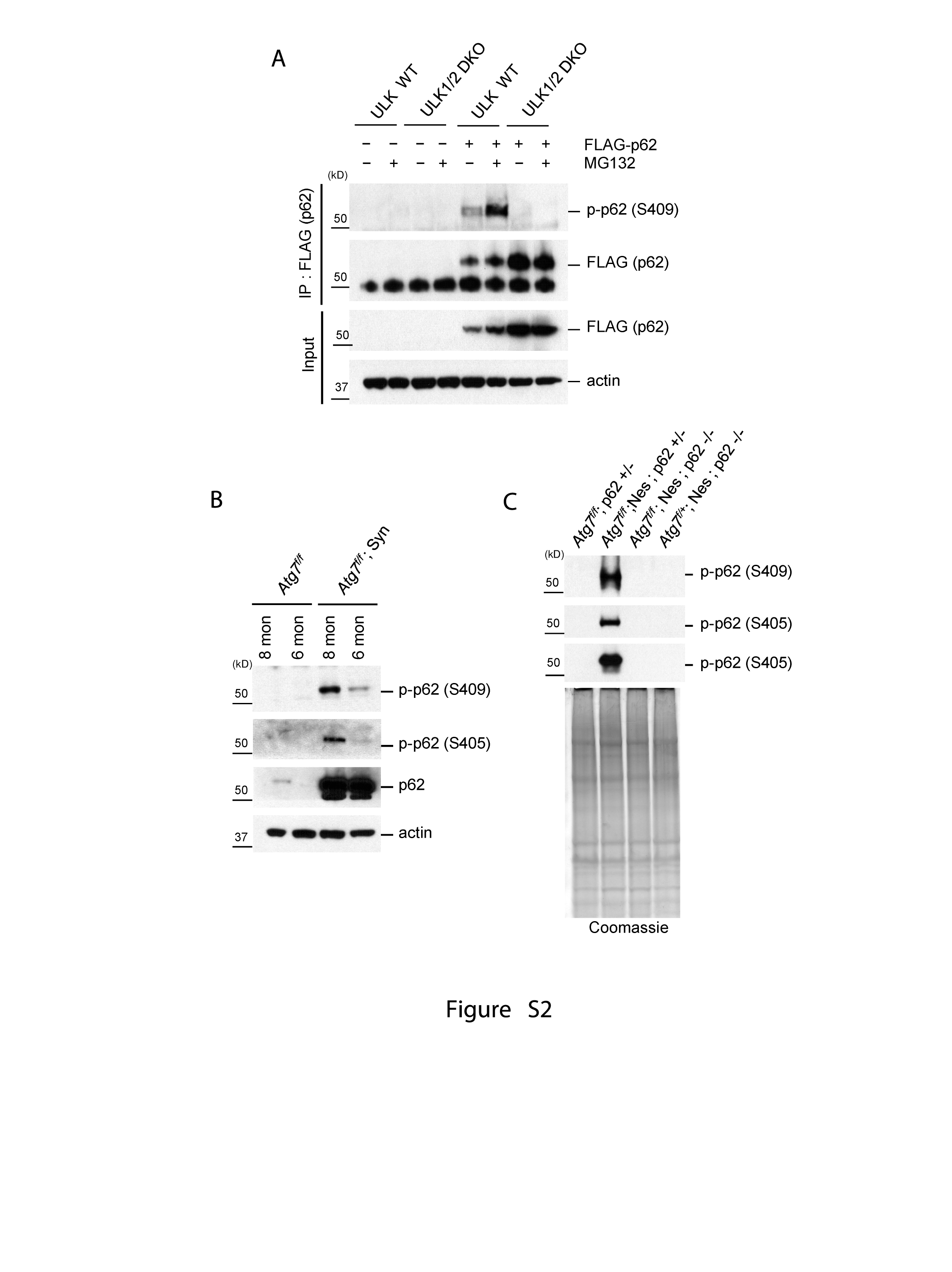

Supplement: S2 Fig — A. ULK1 and ULK2 are responsible for p62 phosphorylation at Ser409 upon MG132 treatment. ULK WT and ULK1/2 double knockout(DKO) MEFs were transfected with empty vector or FLAG-p62 and treated with MG132 treatment. IP with FLAG antibody was performed and immunoprecipitants were analyzed with indicated antibodies. B. The accumulation of p62 p-S409 and p-S405 in autophagy deficient brain tissues. Whole brain lysates of Atg7 f/f and Atg7 f/f; Synapsin-Cre were analyzed. C. Specificity of p-S409 in autophagy deficient brain tissues. Insoluble fraction from whole brain lysates of Atg7 f/f; p62 +/-, Atg7 f/f; nestin-Cre; p62 +/-, Atg7 f/f; nestin-Cre:p62 -/-, Atg7 f/+; nestin-Cre; p62 -/- were immunoblotted with indicated antibodies. The gel was stained with Coomassie blue for loading control. (TIF) [file pgen.1004987.s002.tif]

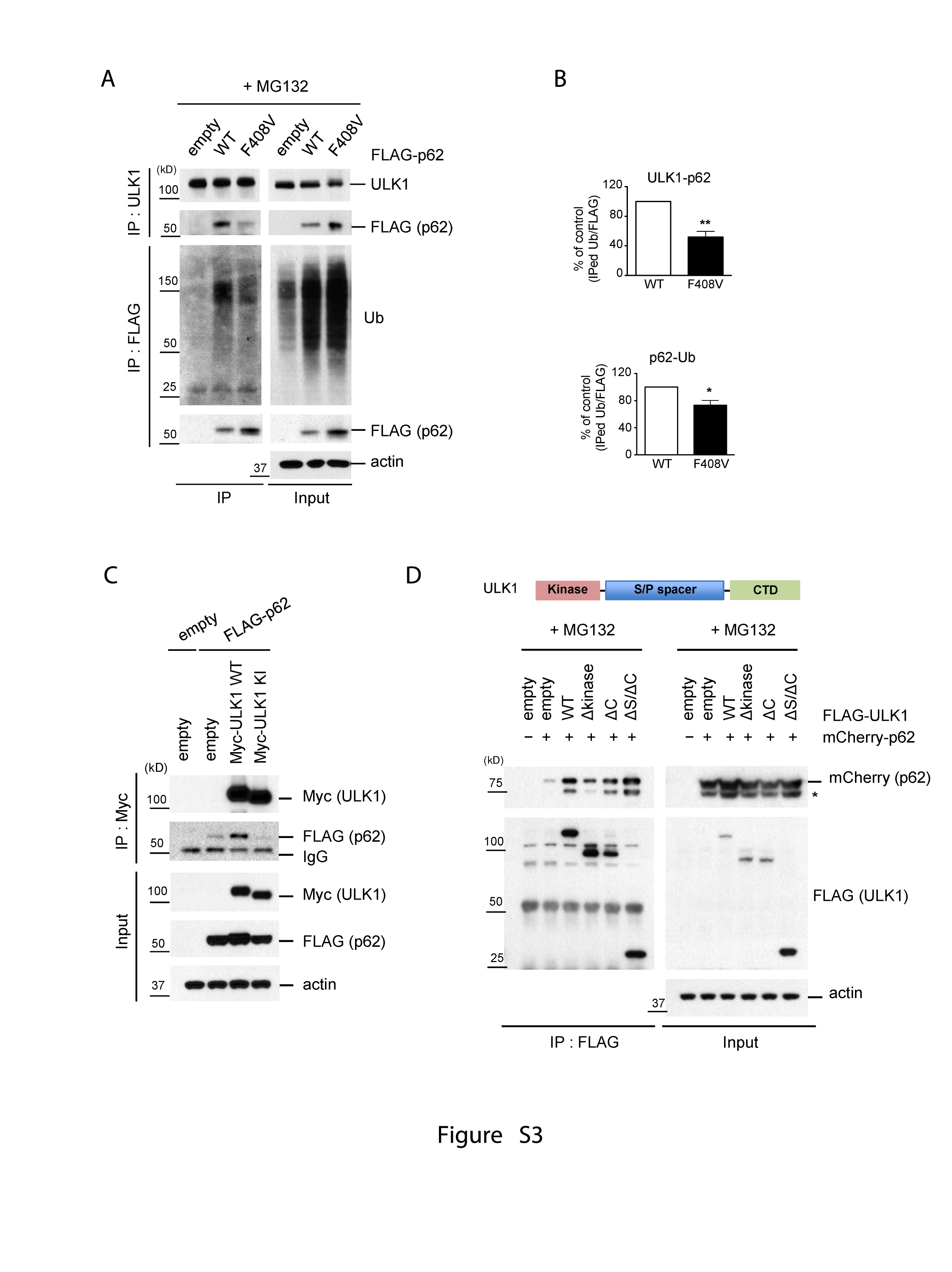

Supplement: S3 Fig — A. ULK1-p62 interaction requires ubiquitin binding site of p62. p62 KO MEFs carrying empty vector, FLAG-p62 WT or F408V mutant were treated with MG132 and cellular lysates were subjected to IP with anti-ULK1 or-FLAG antibodies. Immunoprecipitants were detected with indicated antibodies. B. The level of IPed p62 or ubiquitinated proteins was initially normalized to the level of input and then further normalized to the level of IPed ULK1 or FLAG, respectively. One sample t-test was used and data are represented as mean ± SEM(n = 4). * p < 0.05, ** p < 0.01. C. ULK1 kinase activity is required for p62 binding. HEK 293T cells were transfected with empty vector, Myc-ULK1 WT or KI(kinase inactive mutant) together with FLAG-p62. IP with anti-Myc antibody was performed, followed by immunoblot assay. D. The kinase domain of ULK1 mediates p62 binding. A schematic diagram indicates ULK1 domain structures(Top). HEK 293T cells were transfected with mCherry-p62 WT along with FLAG-ULK1 WT, Δkinase(kinase deletion mutant), ΔC(CTD deletion mutant) or ΔS/ΔC(S/P spacer and CTD deletion mutant). Transfected cells were treated with MG132 and then used in IP with anti-FLAG antibody and analyzed with indicated antibodies. Asterisks indicate non-specific bands. (TIF) [file pgen.1004987.s003.tif]

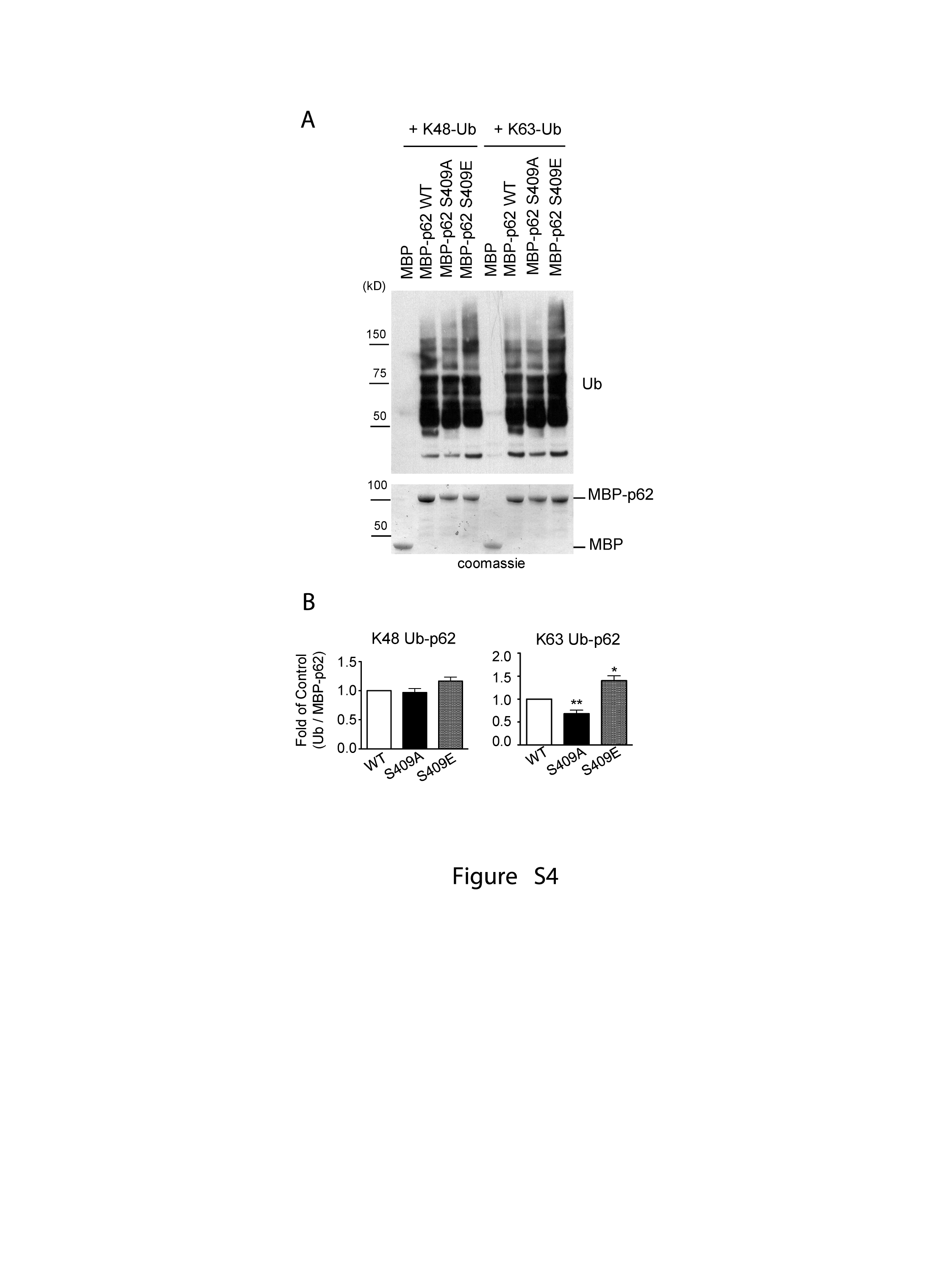

Supplement: S4 Fig — A. P-Ser409 increases p62 and K63-Ub binding. Bacterially expressed MBP, MBP-p62 WT, S409A or S409E were subjected to pull down assay in the presence of K48-or K63-linked ubiquitin peptides. Interaction between Ub and p62 was detected by immunoblotting with Ub antibody(upper panel); MBP and MBP-p62 protein levels were confirmed by coomassie blue gel staining(lower panel). B. Ubiquitin levels pulled down by MBP-p62 S409A or S409E proteins were normalized to the protein input and compared to that of MBP-p62 WT protein. One sample t-test was used and data are represented as mean ± SEM(n = 4). * p < 0.05, ** p < 0.01 (TIF) [file pgen.1004987.s004.tif]

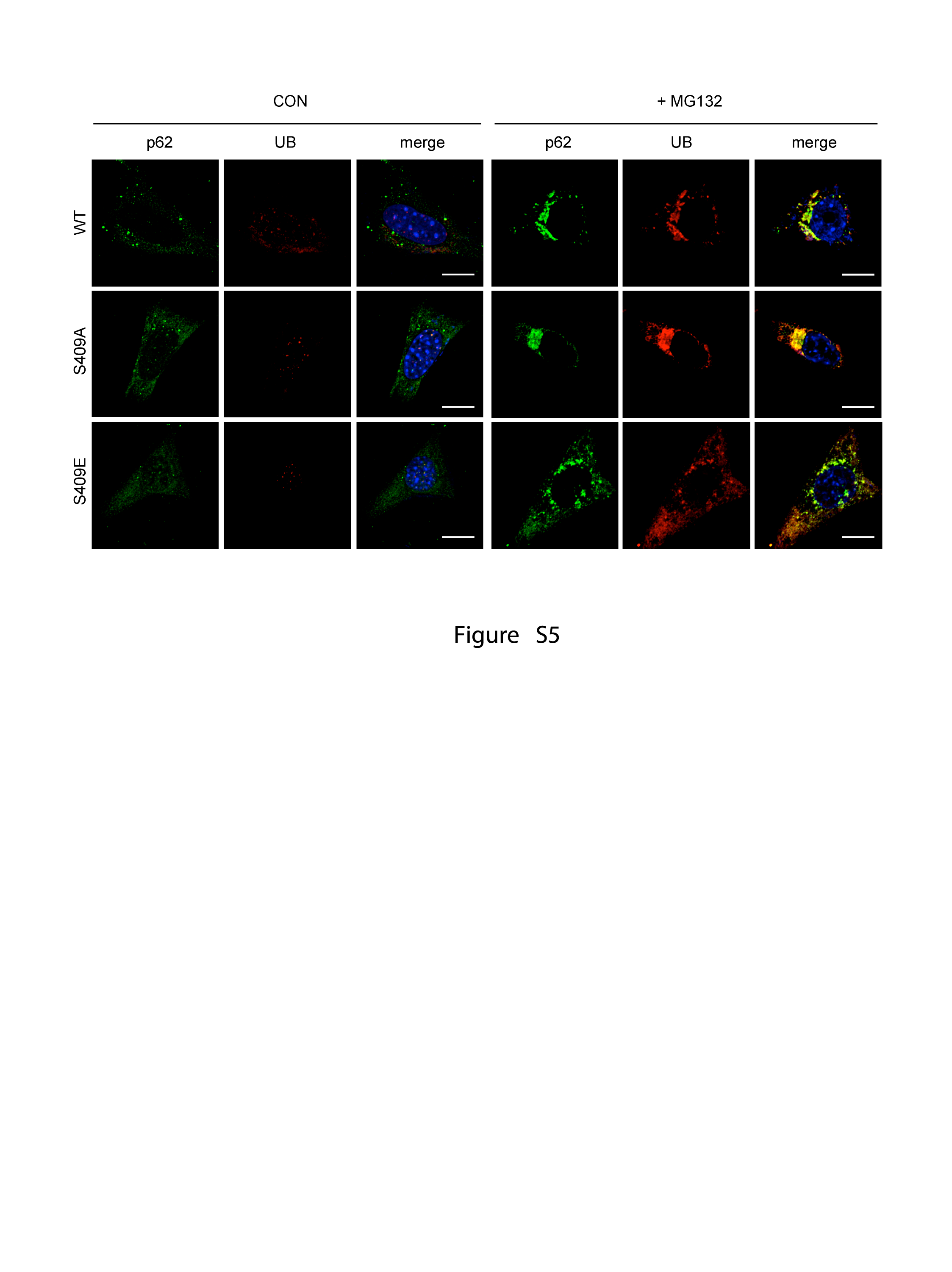

Supplement: S5 Fig — p62 KO MEFs stably over-expressing p62 WT, S409A, or S409E were treated with MG132, fixed, stained with p62(green) and ubiquitin(red) antibodies, and then visualized under fluorescent microscopy. Scale bar = 10 μm. (TIF) [file pgen.1004987.s005.tif]
